# Functional and Safety Profile of *Limosilactobacillus vaginalis* and Development of Oral Fast-Disintegrating Tablets for Gut Microbiota Modulation

**DOI:** 10.3390/pharmaceutics17081011

**Published:** 2025-08-01

**Authors:** Barbara Giordani, Federica Monti, Elisa Corazza, Sofia Gasperini, Carola Parolin, Angela Abruzzo, Claudio Foschi, Antonella Marangoni, Monia Lenzi, Barbara Luppi, Beatrice Vitali

**Affiliations:** 1 Department of Pharmacy and Biotechnology, Alma Mater Studiorum, University of Bologna, 40127 Bologna, Italy; barbara.giordani4@unibo.it (B.G.); federica.monti16@unibo.it (F.M.); elisa.corazza7@unibo.it (E.C.); sofia.gasperini95@gmail.com (S.G.); carola.parolin@unibo.it (C.P.); angela.abruzzo2@unibo.it (A.A.); m.lenzi@unibo.it (M.L.); 2 Section of Microbiology, Department of Medical and Surgical Sciences, Alma Mater Studiorum, University of Bologna, 40127 Bologna, Italy; claudio.foschi2@unibo.it (C.F.); antonella.marangoni@unibo.it (A.M.); 3 Microbiology Unit, IRCCS Azienda Ospedaliero-Universitaria di Bologna, 40138 Bologna, Italy

**Keywords:** *Limosilactobacillus vaginalis*, probiotics, postbiotics, bifidobacteria, fast-disintegrating formulations, freeze-dried tablets, gut health

## Abstract

**Background/Objectives**: Early gut colonization by bifidobacteria, occurring more favorably in vaginally born infants than in those delivered via C-section, is crucial for maintaining overall health. The study investigated the health-promoting properties of *Limosilactobacillus vaginalis* BC17 both as viable cells and as postbiotics (i.e., cell-free supernatant and heat-killed cells), with the purpose of developing oral formulations to support intestinal health. **Methods**: The safety, effects on the adhesion of bifidobacteria and enteropathogens to intestinal cells, and anti-inflammatory properties of *L. vaginalis* BC17 viable cells and postbiotics were evaluated. Fast-disintegrating tablets were formulated by freeze-drying cell-free supernatant in combination with heat-killed or viable cells alongside maltodextrins. **Results**: The formulations were shown to be non-genotoxic and compatible with intestinal cell lines (Caco-2 and HT-29). BC17 viable cells survived in co-culture with intestinal cells up to 48 h and exhibited moderate adhesion to the cell lines. Notably, both BC17 viable cells and postbiotics enhanced the adhesion of beneficial bifidobacteria to Caco-2 cells by up to 250%, while reducing enteropathogens adhesion by 40–70%. Moreover, they exerted significant anti-inflammatory effects, reducing nitric oxide production in macrophages by 40–50% and protecting intestinal cells from SDS-induced damage. The formulations allowed administration of at least 10^9^ BC17 cells in infants and adults through easy and rapid dispersion in milk or water, or directly in the oral cavity without chewing, and preserved their functional properties for up to 3 months of storage. **Conclusions**: *L. vaginalis* BC17 viable cells and postbiotics, as well as fast-disintegrating tablets, showed promising functional and safety profiles. Although further in vivo validation is needed, this approach represents a compelling strategy for promoting gut health.

## 1. Introduction

It is well established that the proper development and functioning of the human body depend on the microbial communities that inhabit it; disruptions to this ecological balance often result in the emergence of infectious or metabolic diseases. In early life, the proper shaping of the intestinal microbiota and the maturation of the immune system are strongly influenced by the interaction between the newborn and the mother’s microbiota, especially the vaginal one during birth and the skin/breast milk microbiota during breastfeeding [1,2]. Indeed, maternal bacteria can be retrieved in infants’ feces and high levels of lactobacilli colonize the mucosae of infants born vaginally [3,4], while babies born by cesarean section or formula-fed generally displayed lower levels of bifidobacteria [5,6,7,8]. In adulthood, overall health, including psychological well-being, has been related to a balanced microbiome fingerprint, which is highly personalized and even unique to each body site [9]. On the other hand, dysbiosis or alterations in the composition of the human microbiota, particularly within the gut, have been linked to the emergence of various diseases, including colitis, cancer and neurodegenerative disorders such as dementia [10,11]. An increasing number of studies highlight the metabolic, structural, and protective roles of microbial communities within the human body including pathogen competition, production of trophic factors, and enhancement of the epithelial barrier [12,13,14]. Probiotics and postbiotics contribute to the prevention of dysbiosis and the restoration of balanced healthy conditions by supplying viable microbial cells or inanimate derivatives with beneficial properties, respectively. Among probiotic microbial strains, *Lactobacillus* and related genera have been largely employed as food supplements or functional food ingredients, due to their recognized antimicrobial and health-promoting activities [15]. Our research group has recently demonstrated that certain beneficial *Lactobacillus* strains of vaginal origin, not only antagonize vaginal pathogens, but also selectively promote the growth of *Bifidobacterium* spp., key beneficial members of the gut microbiota in both infants and adults [16].

In particular, *Limosilactobacillus vaginalis* BC17 strain has shown pronounced bifidogenic properties. Both its cell-free supernatant and heat-killed cells stimulated the growth of bifidobacteria in planktonic and biofilm culture models, and the strain proved to be genetically safe [17]. *Limosilactobacillus* taxa show high prevalence (47.6%) in the human vaginal microbiome, as recently reported in Lebeer et al., 2023 [18]. Despite its predominantly vaginal origin, *L. vaginalis* strains have also been isolated from diverse sources, including fermented foods (e.g., rye sourdough), animal-derived products (e.g., bovine milk), and human milk, and some of these have exhibited probiotic and technological features [19,20,21].

Given these promising findings, a deeper investigation of *L. vaginalis* BC17 and its derivatives is warranted to support their industrial production and effective application in the pharmaceutical/food market. This constitutes the focus of the present study. Here, the genotoxicity profile of the cell-free supernatant and heat-killed cells has been assessed, along with their impact on human cell lines of intestinal origin (i.e., Caco-2 and HT-29 cells) in terms of cell growth and microbial adhesion. Additionally, the anti-inflammatory potential of *L. vaginalis* BC17 derivatives has been explored. To maximize the beneficial effects, cell-free supernatant and viable/heat-killed cells of *L. vaginalis* BC17 have been employed in combination and developed as formulations for infants and adults. Most of the commercially available probiotics are intended for oral delivery, ensuring high consumer acceptance toward probiotic intake [22,23]. They are typically provided as powders in sachets and capsules, or compressed into tablets. However, sachets have limitations in terms of dosage accuracy and require the intake of 1–2 g of powder per dose. Even though powder-filled sachets can also be orodispersible, the large volume associated with such quantities may pose challenges, especially in pediatric applications. Furthermore, sachets are often packaged as single-use doses, raising concerns about sustainability. Regarding capsules and tablets, they represent a significant share of the global market [17] and allow a more accurate and precise dosage than sachets. However, these forms are generally considered unsuitable for children or adults who have swallowing difficulties. Additionally, the technological procedures involved in tableting, namely drying, mixing, and compression, are likely to damage multiple cellular and biologically active components of probiotics [17]. To address these limitations, *L. vaginalis* BC17 and its related postbiotics are formulated as freeze-dried tablets. This approach combines the benefits of solid dosage forms with a formulation process that minimizes damage to sensitive, bioactive components [24]. Furthermore, it can be scaled efficiently from pilot to industrial scale using standard freeze-drying equipment and quality-by-design approaches [25]. The developed formulations have been characterized in terms of physical and bioactive properties, as well as shelf life.

## 2. Materials and Methods

### 2.1. Microorganisms and Culture Conditions

*Limosilactobacillus vaginalis* BC17 was previously isolated from a vaginal swab of a healthy pre-menopausal Caucasian woman, according to the protocol approved by the Ethics Committee of the University of Bologna, Bologna, Italy (52/2014/U/Tess) [26], and deposited in the DSMZ collection (DSM 34059) (Braunschweig, Germany).

*Bifidobacterium* strains (*B. breve* DSM 20091, *B. breve* DSM 20456, *B. bifidum* DSM 20082, *B. bifidum* DSM 20213, *B. bifidum* DSM 20215, *B. longum* subsp. *longum* DSM 20219, *B. longum* subsp. *infantis* DSM 20088, *B. longum* subsp. *infantis* DSM 20090, *B. adolescentis* DSM 20083, *B. adolescentis* DSM 20086, *B. angulatum* DSM 20098) were purchased from DSMZ [16]. *L. vaginalis* and bifidobacteria were routinely cultured in de Man, Rogosa, and Sharpe broth (MRS, Difco, Detroit, MI, USA) supplemented with L-cysteine 0.05% (*w*/*v*) (Merck, Milan, Italy) at 37 °C, in anaerobic jars containing Gas-Pak EZ (Beckton, Dickinson and Co., Milan, Italy).

Enterotoxigenic *E. coli* (ETEC), *Salmonella enterica* serovar typhimurium, and *Yersinia enterocolitica* belongs to the Department of Pharmacy and Biotechnology of University of Bologna and were aerobically grown at 37 °C in Brain Heart Infusion (BHI, Difco) [17,27].

### 2.2. Cell Culture Conditions

Caco-2 cells ATCC^®^ HTB-37^TM^ were routinely grown in Dulbecco’s modified Eagle’s medium (Gibco Life Technologies, Milan, Italy) supplemented with 20% fetal bovine serum (FBS, Gibco Life Technologies) and 1% L-glutamine (Merck) (DMEM complete medium); RAW 264.7 ATCC^®^ TIB-71^TM^ murine macrophages were cultured in DMEM medium supplemented with 10% FBS and 1% L-glutamine; HT-29 ATCC^®^ HTB-37^TM^ cells and TK6 cells were cultured in RPMI-1640 supplemented with 10% FBS and 1% L-glutamine. TK6 cells doubling time is 13 h; cell density should not exceed the critical value of 9 × 10^5^ cells/mL. All cell cultures were incubated at 37 °C in 5% CO_2_.

### 2.3. Preparation of Derivatives from L. vaginalis BC17

To recover derivatives from *L. vaginalis* BC17, an overnight culture of the strain (10 mL) was transferred into 100 mL of MRS broth and allowed to grow for an additional 24 h, reaching a cell density of 1 × 10^9^ colony-forming units (CFU)/mL. The cell pellet and supernatant were then separated by centrifugation (10,000× *g* for 10 min, Centrisart G-16C; Sartorius, Göttingen, Germany). The culture supernatant was adjusted (pH50; Vio Lab Geass, Turin, Italy) to a final pH of 6.5 with NaOH 5M (Merck) and filtered through a 0.22 μm pore-size filter (polyethersulfone 0.22 mm syringe filters; VWR International, Milan, Italy), thus obtaining cell-free supernatant from *L. vaginalis* BC17 (CFS-BC17) [16].

The cell pellet was washed with distilled water and resuspended in sterile saline (NaCl 0.9%, *w*/*v*) at 1 × 10^9^ CFU/mL. Heat-inactivation of *L. vaginalis* BC17 (heat-killed cells, HK-BC17) was achieved by immersing *L. vaginalis* BC17 suspension in a hot water bath (Falc, Bergamo, Italy) at 70 °C for 2 h, followed by cooling at 4 °C for 24 h; these heating and cooling steps were repeated twice. Inactivation was confirmed by plating the *L. vaginalis* BC17 suspension on MRS agar before and after each heat treatment [17].

CFS-BC17 and HK-BC17 derivatives were stored at −20 °C and +4 °C, respectively, until their use.

Three independent batches of each *L. vaginalis* BC17 derivative were prepared and used for testing. To test living cells of *L. vaginalis* BC17 (viable BC17), BC17 was cultured as described above and the cell pellet was resuspended in sterile saline (NaCl 0.9%, *w*/*v*) at 1 × 10^9^ CFU/mL prior to each experiment. Three independent cultures were tested.

### 2.4. Genotoxicity Assay

The genotoxicological assessment was carried out employing the “In Vitro Mammalian Cell Micronucleus Test”, corresponding to the Organisation for Economic Co-operation and Development (OECD) guideline No. 487 [28].

According to this guideline, the test should be conducted on item concentrations that do not exceed cytotoxicity and cytostasis values of 55 ± 5% [28]. Consequently, the concentrations tested should allow cell viability and proliferation rates of at least 45 ± 5% compared to the concurrent negative controls.

Cells were seeded in 24-well culture plates (Corning Inc., Pisa, Italy) at a cell density of 2.5 × 10^5^ cells/mL. The duration of the treatment corresponds to the time required by TK6 cells to complete 1.5–2.0 normal cell cycles. In particular, the short-term experiment included 3 h of treatment with the test item in the presence or absence of an extrinsic source of metabolic activation, i.e., S9 mix (Mutazyme 10% S9 mix, purchased from Moltox, Boone, NC, USA), followed by a 23 h recovery in fresh medium, while the long-term experiment was a continuous 26 h treatment with the test item [28].

#### 2.4.1. Cytotoxicity and Cytostasis Assay on TK6 Cells

Cytotoxicity and cytostasis were measured in samples treated with the following concentrations of CFS-BC17: 0, 0.1, 0.25, 0.5, 1 and 2 μL/mL (corresponding to 0, 0.01, 0.025, 0.05, 0.1, 0.2% *v*/*v*), including the control MRS 2 μL/mL, corresponding to the maximum concentration of MRS reached in cell cultures with the highest CFS-BC17 treatment concentration. Regarding HK-BC17, the concentrations tested were 1 × 10^5^, 2.5 × 10^5^, 5 × 10^5^, 1 × 10^6^, 2 × 10^6^, 1 × 10^7^ HK cells/mL. The last concentration was preliminarily excluded due to an evident precipitate in the cell culture medium.

At the end of the treatments, cytotoxicity and cytostasis were measured using Guava ViaCount Reagent and performing the Guava ViaCount Assay by flow cytometry, according to the manufacturer’s instructions and previous publications [29].

For each sample, the viability percentage and the total number of living cells in the original sample were then automatically calculated by the Guava ViaCount software 4.1 (Cytek Biosciences, Inc., Fremont, CA, USA).

The calculated viability percentage was normalized to the untreated control, which was set at 100%.

Cytostasis was evaluated in terms of Relative Population Doubling (RPD). In particular, based on the number of cells initially seeded and the number detected at the end of the treatment, Population Doubling (PD) was calculated according to the following formula:

$$PD=log\left(\frac{post\text{-}treatment\;cell\;number}{initial\;cell\;number}\right)÷log2$$

Afterward, the RPD was obtained by comparing the PD of the untreated controls and that of the treated samples as follows:

$$RPD=\frac{PD\;in\;treated\;cultures}{PD\;in\;control\;cultures}\times 100$$

#### 2.4.2. Measurement of Micronuclei (MNi) Frequency on TK6 Cells

MNi frequency was measured in samples treated with the three highest concentrations of the test items, that complied with the abovementioned cytotoxicity, cytostasis, and apoptosis thresholds: 0.5, 1, and 2 μL/mL for CFS-BC17 and 5 × 10^5^, 1 × 10^6^, and 2 × 10^6^ cells/mL for HK-BC17. The mutagens mitomycin C (MMC) and vinblastine (VINB) were employed as positive controls for experiments carried out in the absence of S9 mix, while cyclophosphamide (CP) was used for experiments with S9 mix [28]. After the treatment, the MNi frequency was assessed by a flow cytometric protocol developed by Lenzi et al. [29] and already used in previous publications [30,31,32]. The MNi frequency of treated samples was normalized to that of the concurrent untreated controls, set equal to 1, and reported as MNi frequency fold increase. Cytofluorimetric analyses of genotoxicity were all carried out on a Guava EasyCyte 5HT Flow Cytometer II generation system equipped with a class IIIb laser operating at 488 nm (Cytek Biosciences).

### 2.5. Impact of L. vaginalis BC17 and Its Derivatives on Intestinal Cell Viability

The effects of *L. vaginalis* BC17 and its derivatives on the viability of two intestinal cell lines, namely Caco-2 and HT-29 cells, were investigated by MTT assay and erythrosine B exclusion test.

Cells were seeded in 96-well and 24-well culture plates (Corning Inc., Pisa, Italy) for MTT and erythrosine B exclusion assays, respectively, at a cell density of 10^5^ cells/cm^2^ and grown to confluence as described above. Then, the supernatant was removed and Caco-2 or HT-29 cells were treated with viable BC17, CFS-BC17 or HK-BC17 diluted in DMEM or RPMI complete media, respectively. In detail, *L. vaginalis* and its derivatives were applied to intestinal cells (200 μL/well) at a ratio of 100:1 (BC17:Caco-2/HT-29 cells), considering for standardization the initial cell density of the BC17 culture from which CFS-BC17 and HK-BC17 had been derived. The combinations of CFS-BC17 with HK-BC17 (HK+CFS-BC17) or with viable BC17 (viable+CFS-BC17) were also tested. In these cases, CFS-BC17 was applied to the cells at a ratio of 66:1, whilst HK-BC17 and viable BC17 were tested at a ratio of 33:1 each, ensuring that the total amount of *L. vaginalis* BC17 (HK+CFS-BC17 or viable+CFS-BC17) was 100-fold higher than the number of the intestinal cells. Cells treated with MRS at the same volume as CFS-BC17 were also included in the experiment, along with untreated cells used as controls. Plates were incubated for 24 h and 48 h (37 °C, 5% CO_2_). At the end of incubation, the medium was replaced by 100 μL of DMEM or RPMI complete media containing 3-(45-dimethylthiazol-2-yl)-2,5-diphenyltetrazol (MTT) (Merck) at a final concentration of 0.5 mg/mL, and plates were kept in incubation (37 °C, 5% CO_2_) for further 4 h. The formed formazan crystals were dissolved by the addition of 200 μL of isopropanol and quantified by optical density at 570 nm using an EnSpire Multimode Plate Reader (PerkinElmer Inc., Waltham, MA, USA).

For the erythrosine B exclusion viability test, Caco-2 and HT-29 cells were treated as reported for MTT assay and cultured for 24 h and 48 h. Intestinal cells were then detached with trypsin-EDTA (Merck) and diluted 1:2 with erythrosine B solution (0.1% *w*/*v* in phosphate-buffered saline, Merck); living cells were counted in a Bürker chamber (Nikon TMS inverted microscope, Nikon, Tokyo, Japan). All results were expressed as percentages with respect to untreated controls.

### 2.6. Co-Incubation of L. vaginalis BC17 with Intestinal Cells and Survival in Gastric Fluid

Caco-2 and HT-29 cells were seeded in 24-well plates (Corning Inc.) equipped with sterile glass coverslips at a cell density of 5 × 10^4^ cells/cm^2^ and grown up to 70–80% confluence.

Viable BC17 was added to Caco-2 cells applying a ratio of 100:1 (*L. vaginalis* BC17:Caco-2/HT-29 cells); plates were incubated in 5% CO_2_ at 37 °C for 3 h, 24 h or 48 h. At the end of each incubation time (T 3 h, T 24 h, and T 48 h), the adhesiveness and survival of bacterial cells were evaluated.

To determine the adhesion of *L. vaginalis* BC17 to Caco-2 and HT-29, cell monolayers were washed three times with phosphate buffer solution (PBS) pH 7.4, fixed with May-Grünwald and stained with Giemsa as previously reported [27]. Adherent *L. vaginalis* BC17 was counted under an optical microscope (Nikon Eclipse 21, Nikon, 1000× magnification) considering at least 20 randomly chosen fields.

In other wells, after mechanically detaching Caco-2/HT-29 cells with a scraper, the cell suspension was serially diluted in sterile saline and seeded onto MRS agar plates to evaluate the total number of viable *L. vaginalis* BC17 over time. Bacterial viability was also determined before incubation (T0) and after incubation of *L. vaginalis* BC17 in sterile DMEM or RPMI complete medium (T 3 h, T 24 h, and T 48 h), as a reference.

The capacity of *L. vaginalis* BC17 to survive in simulated gastric fluid (SGF) was also evaluated [33]. Briefly, viable BC17 (ca. 10^10^ CFU) was suspended in 30 mL of SGF (125 mmol/L NaCl, 7 mmol/L KCl, 45 mmol/L NaHCO_3_, 3 g/L pepsin, pH 3) and incubated under agitation (100 rpm) in anaerobic conditions to simulate peristalsis for 1.5 h and 3 h. BC17 viability over the time (T0, T 1.5 h and T 3 h) was determined by viable counting on MRS agar plates.

### 2.7. Impact of L. vaginalis BC17 and Its Derivatives on Bifidobacterium spp. and Enteropathogens’ Adhesion to Caco-2 Cells

The effects of *L. vaginalis* BC17 and its derivatives on the adhesion of 11 *Bifidobacterium* strains (listed above), ETEC, *S. enterica*, and *Y. enterocolitica* were evaluated on Caco-2 cells, preventively grown up to 70–80% confluence in 24-well plates. Before the experiments, cells were washed twice with PBS and provided with 1 mL of fresh medium. Test microorganisms were subcultured twice as described in Section 2.1, suspended in sterile saline (1 × 10^9^ CFU/mL), and added to Caco-2 monolayer at ratio 100:1 to mimic localized high bacterial concentration that may occur in close proximity to the intestinal epithelium [34]. Simultaneously, Caco-2 cells were treated with *L. vaginalis* BC17 fractions (viable BC17, MRS/CFS-BC17, and HK-BC17) or the combinations of two (HK+CFS-BC17 and viable+CFS-BC17), using the same ratio (100:1 BC17:Caco-2 cells) described in Section 2.5. Caco-2 cells inoculated only with test microorganisms served as controls. Cells inoculated only with *L. vaginalis* BC17 (ratio 33:1 and 100:1 BC17:Caco-2 cells) or with *L. vaginalis* BC17 coupled with CFS-BC17 (ratio 33:1 viable BC17:Caco-2 cells) were included as additional controls for adhesion experiments. Plates were incubated (37 °C, 5% CO_2_) for 3 h to allow bacterial adhesion. Afterward, the culture medium was removed, and the Caco-2 cells were washed three times with PBS to ensure the removal of non-adherent bacteria. Caco-2 cells were mechanically detached using a scraper and counted with a Bürker chamber. Cell suspensions were serially diluted in sterile saline, and the number of adherent bacteria was determined through viable counts. Specifically, to distinguish between bifidobacteria and viable BC17, *Bifidobacterium* spp. were enumerated on MRS agar plates supplemented with 100 μg/mL mupirocin (AppliChem GmbH, Darmstadt, Germany), while *L. vaginalis* BC17 was counted on MRS agar plates supplemented with 20 μg/mL vancomycin (Merck), plates were incubated at 37 °C under anaerobic conditions. Pathogens were cultured on BHI agar and grown aerobically. Results were expressed as the number of adherent bacteria per Caco-2 cell or as percentages relative to the untreated control.

### 2.8. Anti-Inflammatory Activity of L. vaginalis BC17 and Its Derivatives

The anti-inflammatory potential of *L. vaginalis* BC17 fractions (viable BC17, MRS/CFS-BC17, and HK-BC17) and the combinations of two (HK+CFS-BC17 and viable+CFS-BC17) was investigated in terms of (i) protection from inflammatory stress on intestinal cells, and (ii) reduction of nitric oxide (NO) production in macrophages, as follows.

#### 2.8.1. Protection from Inflammatory Stress

The protection of *L. vaginalis* BC17 and its derivatives on inflammatory stress was evaluated following a protocol adapted from Rocha-Ramírez et al. [35]. Caco-2 and HT-29 cells were seeded in 96-well plates at a cell density of 10^5^ cells/cm^2^ and grown to confluence. DMEM and RPMI complete media supplemented with sodium dodecyl sulfate (SDS, Merck) at 0.05% (*w*/*v*) were used to induce inflammatory stress on Caco-2 and HT-29 cells, respectively. Each one of the samples was added to the well at the ratio 100:1 (*L. vaginalis* BC17:intestinal cells), as reported in Section 2.5. Control wells without SDS and wells exposed to SDS and not treated with samples were also included. Plates were incubated for 24 h or 48 h and the residual cell viability was evaluated by MTT assay, following the same procedure previously described. Results were expressed as percentages with respect to controls not induced with SDS.

#### 2.8.2. Modulation of Nitric Oxide Production

The modulation of NO production was evaluated as reported by Corazza et al. [36]. RAW264.7 macrophages were seeded in 24-well plates (2.5 × 10^5^ cells/cm^2^) and grown for 48 h to reach confluence. Exhaust medium was replaced with 1 mL of fresh DMEM complete medium containing 1 μg/mL lipopolysaccharide (LPS from *E. coli* O55:B5, Enzo Life Sciences (New York, NY, USA), Serotype TLRGRADE) to induce NO production. Cells were simultaneously treated with samples (100:1 ratio *L. vaginalis* BC17:RAW264.7 cells) and incubated for 24 h. Control cells were treated with only LPS. The NO production by macrophages was correlated to nitrite formation in the supernatants, which was quantified by Griess method following the manufacturing procedure of Griess Reagent Nitrite Measurement Kit (Cell Signaling Technology, Milan, Italy). Briefly, after incubation, 100 μL of the culture medium was mixed with 100 µL of Griess reagent, and nitrite concentration was measured spectrophotometrically at 550 nm using an EnSpire Multimode Plate Reader. Results were expressed as percentages relative to control (100%).

### 2.9. Formulation of L. vaginalis BC17 and Its Derivatives in Fast-Disintegrating Tablets

#### 2.9.1. Preparation of Freeze-Dried Tablets

Freeze-dried tablets were prepared using maltodextrin (MX) (Fagron, Quarto Inferiore, BO, Italy) and CFS-BC17 as excipients, whose working concentrations were selected following a screening procedure. Specifically, unloaded tablets (without HK-BC17 or viable BC17) were developed by using only 30% (*w*/*w*) MX or CFS-BC17, otherwise employing MX in the concentration range of 10–30% (*w*/*w*) together with CFS-BC17 either undiluted or diluted 1:2 in sterile water. When the excipients were tested separately, MX was solubilized in sterile water and gently stirred (Arex 5 digital, VELP Scientifica, Usmate Velate, MB, Italy) for 60 min at room temperature (22 ± 2 °C) to remove air bubbles. CFS-BC17 was collected as described in Section 2.3 and either used in its native form or after dilution. Tablets resulting from combining the two excipients were obtained by solubilizing MX in the undiluted or diluted CFS-BC17, followed by stirring. 0.7 g of all unloaded formulations were placed into each cavity (15 mm in diameter) of a blister pack (Farmalabor, Canosa di Puglia, BT, Italy), frozen overnight at −20 °C, freeze-dried at 0.4 mbar and −60 °C for 24 h (Freeze Dryer ALPHA 1–2 LSC Basic, Christ, Milan, Italy), and stored in a desiccator at room temperature until use.

Cell-containing tablets were prepared according to the procedure described above but using 42 g of the selected excipient composition (30% MX in undiluted CFS-BC17) to resuspend 3 × 10^11^ cells of HK-BC17 or viable BC17 (Tab HK+CFS-BC17 and Tab viable+CFS-BC17, respectively), thus resulting in 5 × 10^9^ BC17 cells every 0.7 g of cell suspension (7.1 × 10^9^ cells/g). The latter was poured into the blister packs (0.7 g/cavity), frozen, freeze-dried, and finally stored in a desiccator at room temperature until use.

#### 2.9.2. Size, Weight, Disintegration, pH, and Content Uniformity of the Tablets

Unloaded and cell-containing freeze-dried tablets were characterized in terms of thickness, diameter, and weight. Size attributes were determined through an electronic digital caliper (art. 1367 E 2900; Shanghai ShangErBo Import & Export Company, Shanghai, China), whereas the weight was measured by using an electronic balance (Sartorius ED221S, Muggiò, MI, Italy).

For Tab HK+CFS-BC17 and Tab viable+CFS-BC17, the disintegration time, the pH upon dispersion, and the content uniformity were also evaluated. The disintegration time was recorded using a stopwatch and corresponds to the seconds required for the complete disintegration of the freeze-dried tablet upon contact with 200 mL of the following media: (i) distilled water at 25 °C, (ii) PBS pH 6.8 (8.38 mM Na_2_HPO_4_• 12H_2_O; 7.35 mM KH_2_PO4; 93.94 mM NaCl, salts were from Merck) at 37 °C, and (iii) formula (28.87 g of powdered milk, Coop Italia S.C., Casalecchio di Reno, BO, Italy) in water at 37 °C.

To ensure that the final formulations maintained a pH close to that of the buccal cavity, the parameter was evaluated (Basic20 pH, Crison, Modena, BO, Italy) upon tablet disintegration in 200 mL of water. The content uniformity of tablets was assessed by turbidimetric analysis (UV-Vis spectrophotometer UV-1601, Shimadzu, Milan, MI, Italy) at 600 nm of 10 mL of water in which a single tablet had been dispersed. Each measurement was blank-subtracted using an empty tablet (containing both MX and CFS-BC17, but no cells).

#### 2.9.3. Viability of *L. vaginalis* BC17 and Microbial Contamination Control

Viability of *L. vaginalis* BC17 formulated in Tab viable+CFS-BC17 was evaluated by spotting on MRS agar plates 10 µL of tenfold dilutions of tablets resuspended in 5 mL of sterile saline solution. Plates were incubated at 37 °C in anaerobiosis for 24 h and results were expressed as CFU/tablet.

To assess microbial contamination, Tab HK+CFS-BC17 and Tab viable+CFS-BC17 dispersed in 5 mL of sterile saline were seeded (100 μL) on three different media: Nutrient Agar (NA), Trypticase Soy Agar (TSA), and Sabourad Dextrose Agar (SDA) (Becton, Dickinson and Co., Sparks, NV, USA). NA and TSA plates were incubated in aerobiosis at 35 °C for 72 h, whilst SDA plates were aerobically incubated at 25 °C for 5 days to search for bacterial and fungal contamination, respectively.

#### 2.9.4. Technological and Functional Stability

Tab HK+CFS-BC17 and Tab viable+CFS-BC17 were stored in a desiccator at room temperature and monitored for technological and functional stability over 3 months.

Technological stability evaluations were performed monthly and comprised size and weight measurements according to the procedures described in Section 2.9.2, along with assessments of cell viability and microbial contamination, which were conducted before and after the freeze-drying process (T = 0 days) and at fixed time-points (7, 14, 30, 60, 120 days) following the method reported in Section 2.9.3.

For the functional characterization, tablets were dispersed in 5 mL of sterile saline and tested immediately after preparation and after 3 months of storage in terms of: (i) impact on Caco-2 and HT-29 cell viability; (ii) impact on *Bifidobacterium* spp. (*B. bifidum* DSM 20082, *B. adolescentis* DSM 20086, *B. infantis* DSM 20090, *B. breve* DSM 20091, and *B. longum* DSM 20219) and enterotoxigenic *E. coli* adhesion to Caco-2 cells; (iii) anti-inflammatory activities, by following the same procedures described in Section 2.5, Section 2.7 and Section 2.8, respectively.

### 2.10. Data and Statistical Analysis

The experimental conditions in genotoxicity assay were tested at least in duplicate on independent experiments (*n* = 2), and all assays were performed in at least two independent experiments. Results are expressed as the mean ± Standard Error of the Mean (SEM) and statistical significance was analyzed by paired analysis of variance for repeated measures (repeated measures ANOVA), followed by Dunnett’s correction for multiple comparisons.

**Figure 3:**
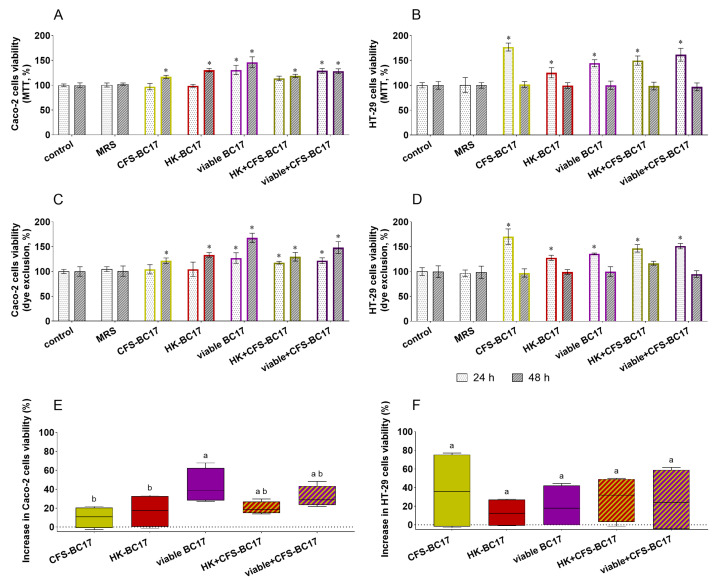
Viability of intestinal cells treated with *L. vaginalis* BC17 fractions (CFS-BC17, HK-BC17, viable BC17, HK+CFS-BC17, and viable+CFS-BC17) for 24 h and 48 h. (**A**) Caco-2 and (**B**) HT-29 viability evaluated by MTT assay; (**C**) Caco-2 and (**D**) HT-29 viability evaluated by dye exclusion assay. Results are reported in percentage compared to the control (100%) (mean ± SD, *n* = 3), * *p* < 0.05 vs. control. The results of the assays are summarized in boxplot reported as increase in cell viability (%) compared to the control (dotted line) for (**E**) Caco-2 cells and (**F**) HT-29 cells. Each box represents the interquartile range (25–75th percentile). Lines within the boxes indicate the median values of the samples. The extremes of the bars indicate the minimum and maximum values, respectively. Statistically significant differences between the samples are indicated by different letters (*p* < 0.05).

**Figure 5:**
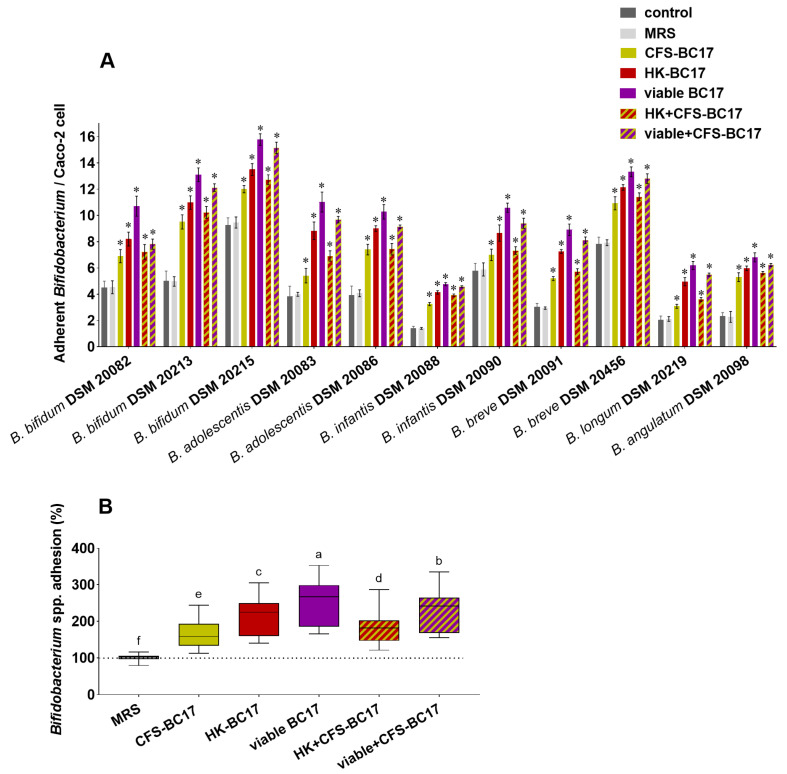
Effect of *L. vaginalis* BC17 fractions (viable BC17, CFS-BC17, HK-BC17, viable+CFS-BC17 and HK+CFS-BC17) on *Bifidobacterium* spp. adhesion to Caco-2 cells. (**A**) Adherent *Bifidobacterium* per Caco-2 cell after 3 h of co-incubation (mean ± SD, *n* = 3), * *p* < 0.05 (vs. control). (**B**) Boxplot of *Bifidobacterium* adhesion percentage compared to the control (100%). Each box represents the interquartile range (25–75th percentile). Lines within the boxes indicate the median values of the samples. The extremes of the bars indicate the minimum and maximum values, respectively. Statistically significant differences between the samples are indicated by different letters (*p* < 0.05).

**Figure 7:**
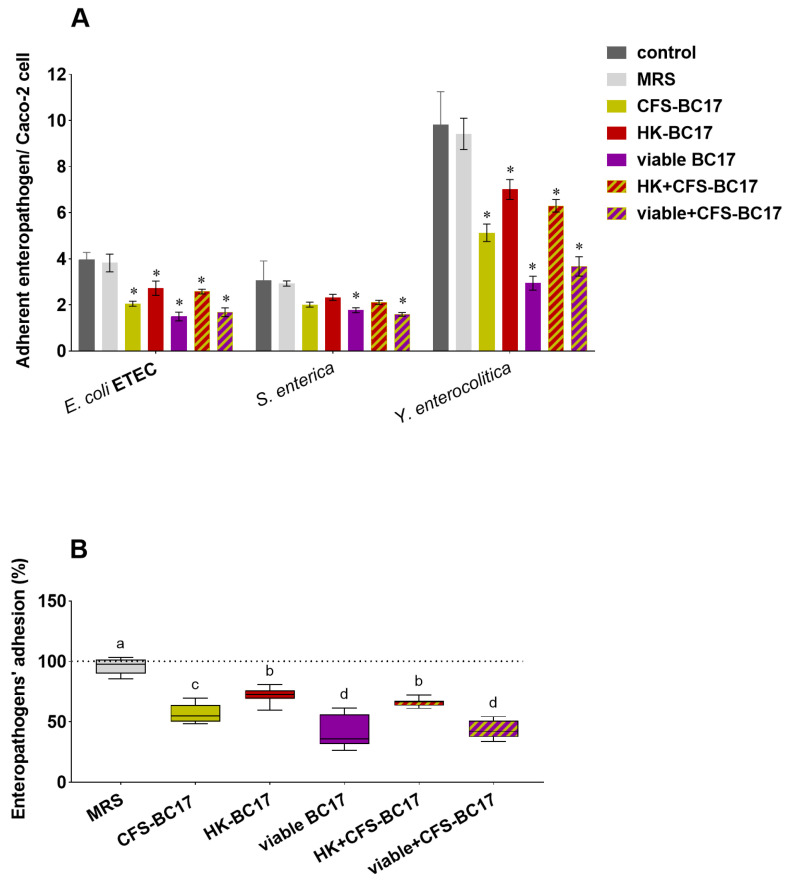
Effect of *L. vaginalis* BC17 fractions (viable BC17, CFS-BC17, HK-BC17, viable+CFS-BC17 and HK+CFS-BC17) on enteropathogens adhesion to Caco-2 cells. (**A**) Adherent enterotoxigenic *E. coli* (ETEC), *S. enterica* and *Y. enterocolitica* per Caco-2 cell after 3 h of co-incubation (mean ± SD, *n* = 3), * *p* < 0.05 (vs. control). (**B**) Boxplot of enteropathogen adhesion percentage compared to the control (100%). Each box represents the interquartile range (25–75th percentile). Lines within the boxes indicate the median values of the samples. The extremes of the bars indicate the minimum and maximum values, respectively. Statistically significant differences between the samples are indicated by different letters (*p* < 0.05).

**Figure 12:**
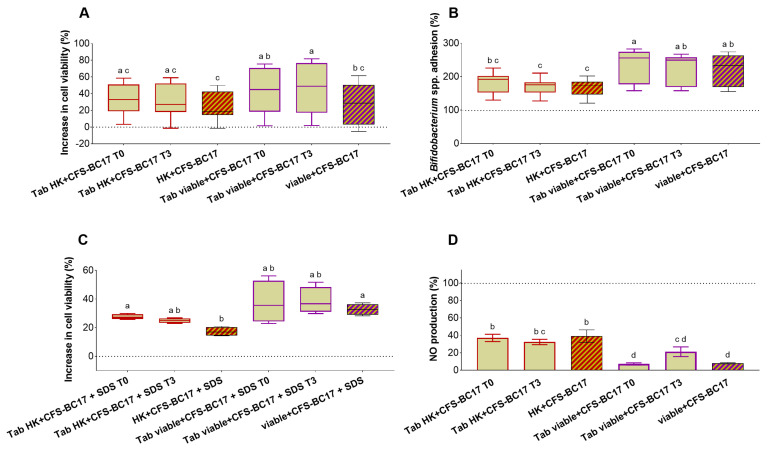
Functional characterization of tablets (Tab HK+CFS-BC17 and Tab viable+CFS-BC17). Tablets were tested immediately after the preparation (T0) and after 3 months of storage at room temperature (T3). (**A**) Effects on viability of Caco-2 and HT-29 cells after 24 h and 48 h of incubation, evaluated by MTT assay and dye exclusion test. Overall results are presented as boxplot reporting the increase of cell viability (%) compared to the control (dotted line). (**B**) Effects on *Bifidobacterium* spp. adhesion to Caco-2 cells. Boxplot reported the percentages compared to the control (100%). (**C**) Protective effects on Caco-2 and HT-29 cells exposed to SDS 0.05% for 24 h and 48 h. Overall results are presented as boxplot reporting the increase of cell viability (%) compared to cells exposed to SDS (dotted line). Each box represents the interquartile range (25–75th percentile). Lines within the boxes indicate the median values of the samples. The extremes of the bars indicate the minimum and maximum values, respectively. (**D**) Inhibitory effect on NO production in LPS-induced macrophages. Results are in percentages with respect to control (means ± SD). All tests were performed in triplicate; statistically significant differences between the samples are indicated by different letters (*p* < 0.05).

All the other experiments were performed at least in triplicate on independent experiments (*n* = 3) and results were expressed as mean ± Standard Deviation (SD). Two-way ANOVA followed by Dunnett’s correction for multiple comparisons was applied to analyze data of MTT assay, erythrosine B exclusion test, adhesion assays, and protection from SDS stress test. The significance of the differences in the modulation of NO production, co-incubation of *L. vaginalis* BC17 with Caco-2 cells and evaluation of formulated *L. vaginalis* BC17 viability experiments was assessed by applying ordinary one-way ANOVA followed by Dunnett’s correction for multiple comparisons. Repeated measures one-way ANOVA followed by Tukey’s correction for multiple comparisons was used to compare datasets in boxplots in Figure 3E,F, Figure 5B, Figure 7B,C and Figure 12A–C.

All statistical analyses were performed using GraphPad Prism version 10.3.1 for Windows (GraphPad Software, San Diego, CA, USA) and differences were deemed significant for *p* values below 0.05.

## 3. Results

### 3.1. Assessment of the Genotoxicity of L. vaginalis BC17 Derivatives

**Figure 1:**
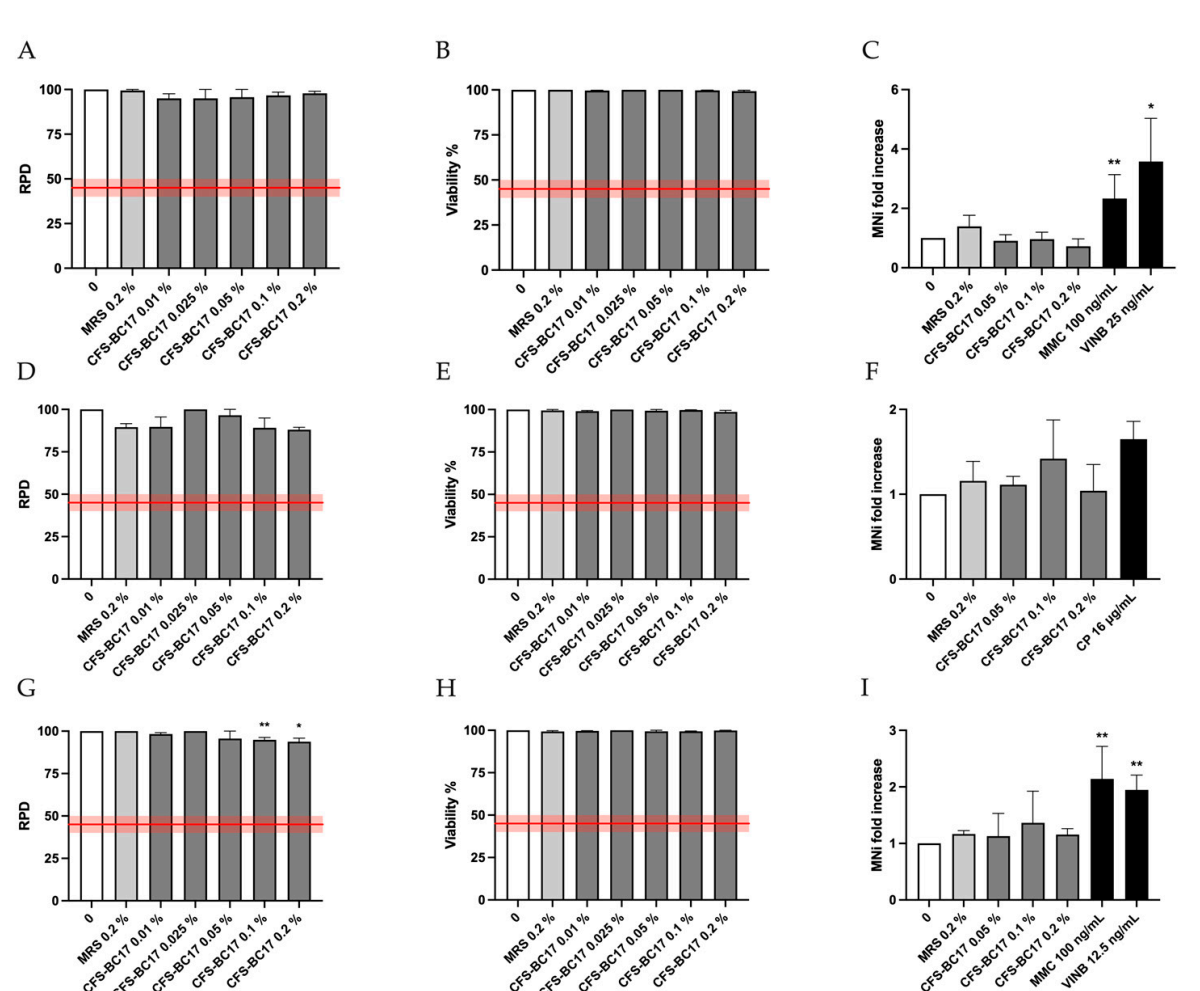
Genotoxicity assay on CFS-BC17. RPD, cell viability%, and MNi-fold increase of TK6 cells after 3 h treatment without S9 mix (**A**–**C**), 3 h treatment supplemented with S9 mix (**D**–**F**) or 26 h treatment (**G**–**I**) at the indicated concentrations of CFS-BC17 and compared to the concurrent untreated control (0), control MRS 0.2% *v*/*v* or positive controls (MMC, VINB, CP). The red lines represent the OECD threshold for RPD and viability (45 ± 5%). Results are reported as the mean ± SEM (*n* = 2). * *p* < 0.05 vs. (0); ** *p* < 0.01 vs. (0).

The results of the Guava ViaCount assay on TK6 cells treated with CFS-BC17 showed that all the concentrations tested under three test conditions (3 h ± S9 mix followed by 23 h recovery and 26 h) complied with the thresholds for the cytostatic (RPD) and cytotoxic (viability) effects established by OECD guideline No. 487 for the “In Vitro Mammalian Cell Micronucleus Test” [28] corresponding to 45 ± 5% of the concurrent negative controls (Figure 1A,B,D,E,G,H).

Based on these results, the three highest concentrations were selected for the MNi test.

The results show that the fold increases in MNi frequency in the samples treated with CFS-BC17 were not statistically significant at any of the treatment conditions, indicating that CFS-BC17 is not genotoxic with respect to the induction of structural and numerical chromosomal aberrations. Indeed, after 3 h of treatment in the absence of metabolic activation (S9 mix), the MNi frequency fold increase remained comparable to that of the concurrent negative controls (Figure 1C). After 3 h of treatment in the presence of metabolic activation S9 mix (Figure 1F), and after the long-term treatment of 26 h in the absence of metabolic activation S9 mix (Figure 1I), some fluctuations in the results were observed but none of them were statistically significant; therefore, they can be considered negligible.

**Figure 2:**
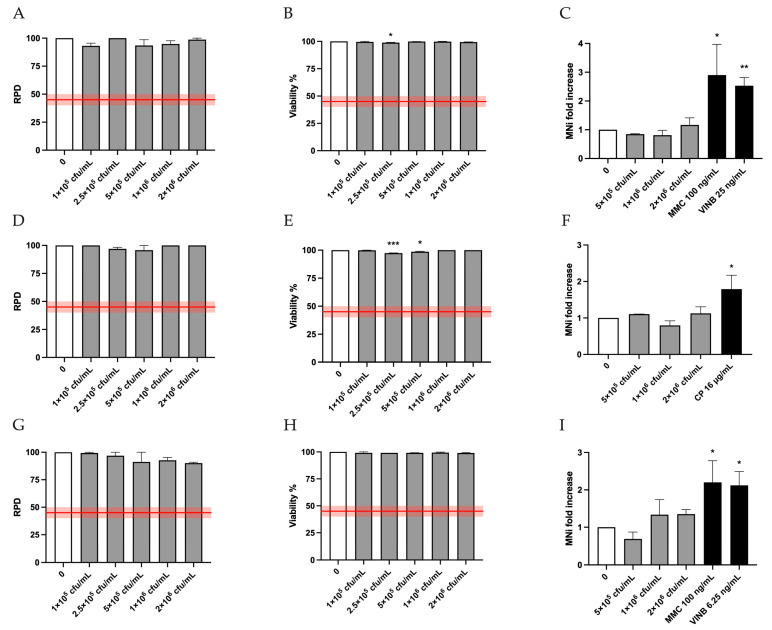
Genotoxicity assay on HK-BC17. RPD, cell viability%, and MNi fold increase of TK6 cells after 3 h treatment without S9 mix (**A**–**C**), 3 h treatment supplemented with S9 mix (**D**–**F**) or 26 h treatment (**G**–**I**) at the indicated concentrations of HK-BC17 and compared to the concurrent untreated control (0% *v*/*v*) or to the positive controls (MMC, VINB, CP). The red lines represent the OECD threshold for RPD and viability (45 ± 5%). Results are reported as the mean ± SEM (*n* = 2). * *p* < 0.05 vs. (0); ** *p* < 0.01 vs. (0); *** *p* < 0.001 vs. (0).

Analogously, the results of the Guava ViaCount assay on TK6 cells treated with HK-BC17 showed that all the concentrations tested under the three test conditions (3 h ± S9 mix followed by 23 h recovery and 26 h) complied with the thresholds for the cytostatic (RPD) and cytotoxic (viability) effects established by OECD guideline No. 487 for the “In Vitro Mammalian Cell Micronucleus Test” [28], which correspond to 45 ± 5% of the concurrent negative controls (Figure 2A,B,D,E,G,H). Based on these results, the three highest concentrations were selected for the MNi test. HK-BC17 was not genotoxic in terms of induction of structural and numerical chromosomal aberrations. Indeed, under the three experimental conditions, the fold increase in the treated cell cultures remained comparable to that of the concurrent negative controls (Figure 2C,F,I).

### 3.2. Compatibility of L. vaginalis BC17 and Its Derivatives with Intestinal Cells

The MTT assay and erythrosine B exclusion test were performed in order to investigate the compatibility of *L. vaginalis* BC17 and its derivatives in the gut environment. For this purpose, Caco-2 cells, representative of enterocytes, and HT-29 cells, representative of goblet cells, were employed as they are two of the major models of intestinal cells [37]. In particular, they were incubated for 24 h or 48 h with living cells of *L. vaginalis* BC17 (viable BC17), with non-living derivatives (i.e., CFS-BC17 and HK-BC17) or with two combinations of these fractions (HK+CFS-BC17 and viable+CFS-BC17), and results are reported in Figure 3. The results collected with the two assays are in agreement with each other (Pearson’s correlation coefficient obtained from a correlation analysis between datasets was found to be 0.9193) and clearly indicated that *L. vaginalis* BC17 fractions did not negatively impact the mitochondrial activity (MTT assay, Figure 3A,B) or cell proliferation (dye exclusion assay, Figure 3C,D) of both Caco-2 and HT-29 cells. On the contrary, in the presence of all *L. vaginalis* BC17 fractions, a stimulatory effect on cell viability and proliferation was observed. This is particularly evident for Caco-2 cells after 48 h of incubation (cell viability ranged from 116% to 146% in MTT assay, and from 122 to 168% in dye exclusion assay, *p* < 0.05) and for HT-29 cells incubated 24 h (cell viability ranged from 125% to 177% in MTT assay, and from 128 to 170% in dye exclusion assay, *p* < 0.05). Instead, no effect was observed in the presence of MRS medium used for *L. vaginalis* BC17 cultivation. The data collected from the two assays were then grouped according to *L. vaginalis* BC17 fraction considered (viable BC17, CFS-BC17, HK-BC17, HK+CFS-BC17 and viable+CFS-BC17) and summarized in boxplots in Figure 3E,F for Caco-2 cells and HT-29 cells, respectively. Looking at Caco-2 cells, the increase in viability was more pronounced in the presence of viable BC17 (median increase of 39%, *p* < 0.05) than when cells were incubated with its derivatives, which showed similar behavior (median cell viability increase of 11% and 17% with CFS-BC17 and HK-BC17, respectively, *p* > 0.05) (Figure 3E). The combinations of CFS-BC17 with viable BC17 or HK-BC17 had an intermediate effect compared to the single fraction (median increase of 18% for HK+CFS-BC17 and of 29% for viable+BC17-CFS, *p* < 0.05). No significant difference was found between HK+CFS-BC17 and viable+CFS-BC17, with the only exception being in MTT assay at 24 h of incubation where viable+CFS-BC17, but not HK+CFS-BC17, significantly increased cell viability by 31% (Figure 3A). HT-29 cell viability was similarly affected by all BC17 fractions considered (median increase of 12–36%) (Figure 3F). However, when looking at a single time point, the viability of HT-29 cells incubated with CFS-BC17 for 24 h was significantly higher (170–177%) compared to the incubation with HK-BC17 (125–128%) or viable BC17 (136–144%). The 24 h treatment with the combination HK+CFS-BC17 (147–150%) resulted in lower viability than that observed with CFS-BC17, but higher than with HK-BC17 (*p* < 0.05). Even in the presence of viable+CFS-BC17 the HT-29 cell viability seemed to be lower than with CFS-BC17 alone and slightly higher than with viable BC17, but the differences were not statistically significant.

### 3.3. Probiotic Properties of L. vaginalis BC17

To be considered a probiotic, a microorganism should survive the passage through the stomach and temporarily colonize the intestinal microenvironment for a sufficient period to exert a beneficial effect on the host. From this perspective, *L. vaginalis* BC17 tolerance to SGF was assessed after 1.5 h and 3 h to mimic the gastric half-emptying time in infants and adults, respectively [38]. The results (T0: 8.38 ± 0.52 log CFU/mL, T 1.5 h: 8.16 ± 0.62 log CFU/mL, T 3 h: 8.10 ± 0.68 log CFU/mL) demonstrated a good intrinsic ability of the strain to survive in an acidic environment, suggesting that, even in the absence of a gastro-resistant formulation, at least 10^8^ CFU are capable of reaching the site of action, as required by the FAO guidelines [39] for a probiotic dose.

Moreover, to evaluate the adhesion and survival of *L. vaginalis* BC17 in the presence of intestinal cells, the strain was co-cultured with Caco-2 and HT-29 cells for short (3 h) and prolonged (24 h and 48 h) incubation times.

**Figure 4:**
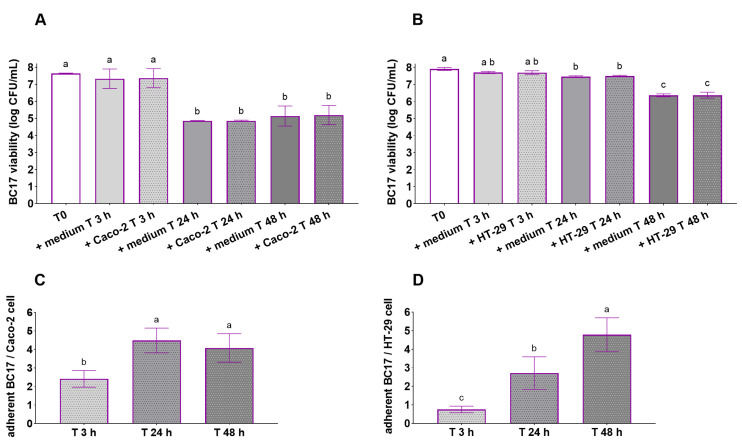
Survival and adhesion of *L. vaginalis* BC17 co-incubated with Caco-2 and HT-29 cells. Viability (log CFU/mL) of *L. vaginalis* BC17 co-incubated with (**A**) Caco-2 (+Caco-2) or without cells (+medium), and with (**B**) HT-29 (+HT-29) or without cells evaluated prior to incubation (T0) and after 3 h, 24 h or 48 h of incubation. Adhesion of *L. vaginalis* BC17 to (**C**) Caco-2 cells (adherent BC17/Caco-2 cell), and (**D**) HT-29 cells (adherent BC17/HT-29 cell), after 3 h, 24 h or 48 h of incubation. All data are reported as mean ± SD (*n* = 3). Statistically significant differences between the samples are indicated by different letters (*p* < 0.05).

The viability of *L. vaginalis* BC17 prior to incubation (T0, 7.6–7.9 log CFU/mL) and during co-incubation with Caco-2 and HT-29 cells is shown in Figure 4A,B, respectively. After 3 h, the viability of *L. vaginalis* BC17 was preserved both when incubated in the presence of the cell monolayer (7.4 log CFU/mL with Caco-2 and 7.7 log CFU/mL with HT-29 cells) and in the presence of the culture medium alone (7.3 log CFU/mL with DMEM and 7.8 log CFU/mL with RPMI) (*p* > 0.05 vs. T0). Overall, no significant difference was found between *L. vaginalis* BC17 incubated alone or with Caco-2/HT-29 cells, indicating that the presence of intestinal cells in co-culture did not affect the viability of *L. vaginalis* BC17. Therefore, the decrease in *L. vaginalis* BC17 viability observed during prolonged incubation with Caco-2 cells (a 2.5 log CFU/mL reduction after 24 h) was not attributable to cellular metabolites in the culture medium but likely reflected a physiological decline due to *L. vaginalis* BC17 not being incubated under its optimal growth conditions. After 48 h, the bacterial count remained stable (5.2 log CFU/mL) with no further decline in viability compared to T 24 h (Figure 4A). The behavior in the presence of HT-29 cells was slightly different: *L. vaginalis* BC17 viability was preserved from 3 h to 24 h of incubation (7.5 log CFU/mL, *p* > 0.05 vs. T 3 h), but significantly diminished after 48 h (6.4 log CFU/mL) (Figure 4B).

The adhesion of *L. vaginalis* BC17 to Caco-2 and HT-29 cells is reported as the number of adherent bacteria per cell in Figure 4C,D. After 3 h of incubation, *L. vaginalis* BC17 showed a moderate ability to adhere to Caco-2 cells (2.4 adherent BC17/Caco-2 cell) and poor adhesion to HT-29 cells (<1 adherent BC17/HT-29 cell). Interestingly, despite the reduction in viability reported above, the adhesion of *L. vaginalis* BC17 to intestinal cells significantly increased after 24 h of incubation with both Caco-2 cells (4.5 adherent BC17/Caco-2 cell) and HT-29 cells (2.7 adherent BC17/HT-29 cell). Adhesion to Caco-2 cells remained higher compared to T0 even after 48 h of incubation (4.1 adherent BC17/Caco-2 cell, *p* < 0.05), although it slightly decreased relative to T 24 h (*p* > 0.05). Conversely, adhesion to HT-29 cells increased from 24 h to 48 h of incubation (*p* < 0.05), reaching 4.8 adherent bacteria per cell.

### 3.4. Effects of L. vaginalis BC17 and Its Derivatives on the Adhesion of Bifidobacterium spp. and Enteropathogens to Caco-2 Cells

Caco-2 cells were used as an epithelial cellular model to assess the capacity of *L. vaginalis* BC17 fractions alone (viable BC17, CFS-BC17, HK-BC17) or in combination (HK+CFS-BC17 and viable+CFS-BC17) to modulate the adhesion of beneficial *Bifidobacterium* spp. (i.e., *B. bifidum*, *B. adolescentis*, *B. infantis*, *B. breve*, *B. longum*, *B. angulatum*) and enteropathogens (i.e., Enterotoxigenic *E. coli*, *Salmonella enterica* and *Yersinia enterocolitica*) after 3 h of co-incubation. The adhesion of *L. vaginalis* BC17 in the presence of test microorganisms (*Bifidobacterium* spp. or enteropathogens) was also determined.

#### 3.4.1. *Bifidobacterium* spp. Adhesion in the Presence of *L. vaginalis* BC17 and Its Derivatives

First, *L. vaginalis* BC17 fractions were sought for stimulating effects on the adhesion of bifidobacteria, and the data are reported in Figure 5. The ability of bifidobacteria alone (control) to adhere to Caco-2 cells varied greatly depending on the *Bifidobacterium* strain, with *B. bifidum* DSM 20215 being the most adhesive (9.4 adherent bacteria/cell) and *B. infantis* DSM 20088 being the least adhesive (<2 adherent bacteria/cell). However, the adhesion of all bifidobacteria was significantly improved when incubated in the presence of each of *L. vaginalis* BC17 fractions, reaching nearly 16 adherent bacteria per Caco-2 cell in the case of *B. bifidum* DSM 20215 co-incubated with viable BC17 (Figure 5A).

To identify possible differences in stimulating activity among *L. vaginalis* BC17 fractions, the data were grouped into a boxplot in Figure 5B as percentages compared to the control. Viable BC17 was the most effective fraction in promoting *Bifidobacterium* spp. adhesion, allowing for adhesion percentages ranging from 166% and 353% (median value of 267%). The association of living *L. vaginalis* BC17 cells with its supernatant (viable+CFS-BC17) also effectively promoted the adhesion of bifidobacteria to Caco-2 cells, with percentages only slightly lower (156–334%, *p* < 0.05) than those observed with viable BC17. The adhesion of *Bifidobacterium* spp. in the presence of viable+CFS-BC17 was in turn higher (*p* < 0.05) than in the presence of CFS-BC17. However, CFS-BC17 had a positive impact on the capacity of all bifidobacteria strains to adhere to Caco-2 cells, and adhesion percentages were variable from 113% to 244%. No effect was observed in the presence of MRS medium.

Additionally, a substantial increase in *Bifidobacterium* spp. adhesion was observed in the presence of non-living *L. vaginalis* BC17 cells (HK-BC17). Overall, the adhesion percentages (140–305%) were lower than those obtained with viable BC17 (Figure 5B), but substantially higher (*p* < 0.05) than those achieved with CFS-BC17. This suggests that the cellular component of *L. vaginalis* BC17 (whether viable or heat-inactivated) is more efficient in mediating *Bifidobacterium* spp. adhesion than the cell-free supernatant. The combination of *L. vaginalis* BC17 derivatives (HK+CFS-BC17) resulted in intermediate activity (adhesion percentages of 121–287%) compared to the individual fractions, consistent with the findings for viable+CFS-BC17. Interestingly, despite *B. bifidum* DSM 20088 poorly adhered to Caco-2 cells, it was the strain most stimulated by all *L. vaginalis* BC17 fractions tested, as the adhesion increased from 1.4 adherent bacteria/cell to up to 4.8 adherent bacteria/cell when incubated with viable-BC17 (adhesion increase of +253%).

**Figure 6:**
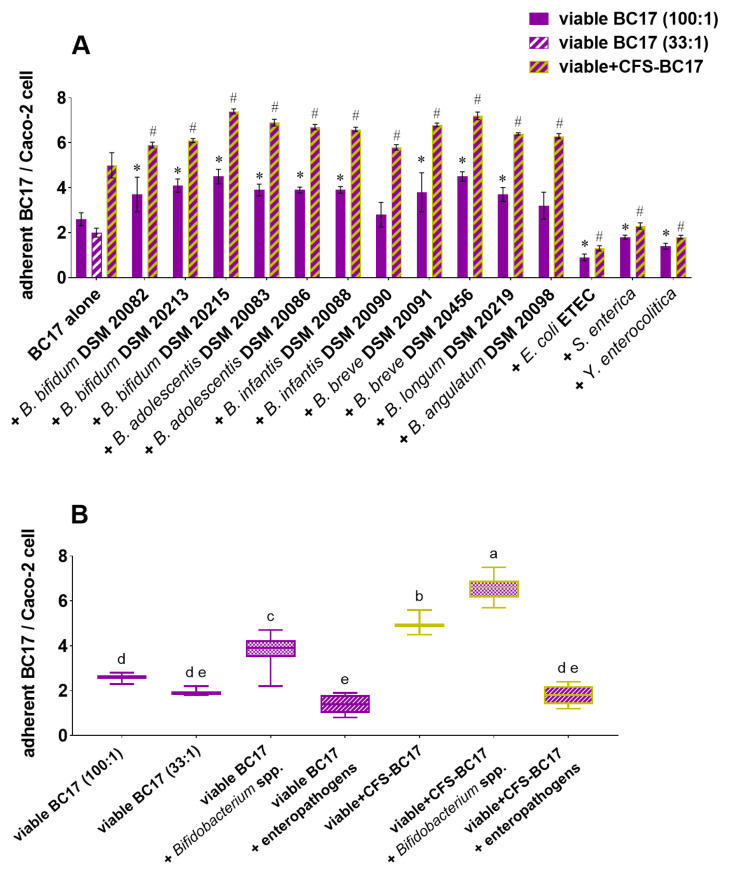
*L. vaginalis* BC17 adhesion to Caco-2 cells co-incubated with *Bifidobacterium* spp. and enteropathogens. The adhesion of *L. vaginalis* BC17 without test microorganisms (BC17 alone) was evaluated for comparison as follows: viable BC17 at ratios of 100:1 and 33:1 with Caco-2 cells, and viable BC17 in the presence of CFS (viable+CFS-BC17). (**A**) Results are reported as adherent BC17 per Caco-2 cell for *L. vaginalis* in the presence of bifidobacteria, enterotoxigenic *E. coli* (ETEC), *S. enterica* or *Y. enterocolitica* (mean ± SD, *n* = 3), * *p* < 0.05 (viable BC17+ test microorganisms vs. BC17 alone 100:1), # *p* < 0.05 (viable+CFS-BC17 + test microorganisms vs. viable+CFS-BC17 alone). (**B**) Boxplot of adherent BC17 per Caco-2 cell. Lines within the boxes indicate the median values of the samples. The extremes of the bars indicate the minimum and maximum values, respectively. Statistically significant differences between the samples are indicated by different letters (*p* < 0.05).

*L. vaginalis* BC17 (viable BC17 and viable+CFS-BC17) adhesion to Caco-2 cells in the presence of bifidobacteria is reported in Figure 6A and summarized according to *L. vaginalis* BC17 fraction as a boxplot in Figure 6B. It is noteworthy that CFS-BC17 not only stimulated the adhesion of bifidobacteria to Caco-2 cells, but also enhanced the adhesion of *L. vaginalis* BC17 itself. When viable BC17 was incubated in the absence of bifidobacteria (BC17 alone, Figure 6A) at a ratio of 100:1 with Caco-2 cells, 2.6 adherent BC17 per Caco-2 cells were found, consistent with the results obtained with May-Grünwald staining (Section 3.3).

However, in the case of incubation with viable BC17+CFS, a ratio of 33:1 (viable BC17:Caco-2 cells) was used to account for the contribution of the supernatant (added to cells at a ratio of 66:1). For this reason, the adhesion of *L. vaginalis* BC17 alone at a ratio of 33:1 was investigated as well and found to be only slightly lower (*p* > 0.05, Figure 6B) than when a ratio of 100:1 was applied (2.0 adherent BC17/Caco-2 cell), thus confirming the capacity of *L. vaginalis* BC17 to adhere to intestinal cells. Notably, the adhesion of *L. vaginalis* BC17 to Caco-2 cells concurrently treated with CFS-BC17 turned out to be more than doubled in the absence of bifidobacteria (5.0 adherent BC17 per Caco-2 cell, *p* < 0.05). Moreover, co-incubation of viable+CFS-BC17 with each of 11 *Bifidobacterium* strains considered further increased the adhesion of *L. vaginalis* BC17 to Caco-2 cells (5.7–7.5 adherent BC17 per Caco-2 cell, *p* < 0.05). The positive impact of bifidobacteria on *L. vaginalis* BC17 was also evident when comparing the adhesion of viable BC17 co-incubated with bifidobacteria (2.8–4.5 adherent BC17 per Caco-2 cell) to the adhesion of viable BC17 incubated alone at a ratio of 100:1.

#### 3.4.2. Enteropathogens’ Adhesion in the Presence of *L. vaginalis* BC17 and Its Derivatives

*L. vaginalis* BC17 fractions were also tested against gut pathogens, and undesired stimulating effects were excluded, as shown in Figure 7. In contrast, all *L. vaginalis* BC17 fractions exhibited a significant anti-adhesive effect on ETEC and *Y. enterocolitica*, reducing ETEC adhesion from 4.0 to 1.5–2.7 adherent bacteria per cell and *Y. enterocolitica* adhesion from 9.8 to 2.9–7.0 adherent bacteria per cell (Figure 7A). However, differences in activity among *L. vaginalis* BC17 fractions were observed, with viable BC17 and viable+CFS-BC17 being the most active samples, as they were the only ones able to significantly inhibit *S. enterica* adhesion (from 3.1 to 1.6–1.8 adherent bacteria per cell). The boxplot in Figure 7B shows that viable BC17, in addition to exerting the highest bifidogenic effect, was also the most efficient in reducing pathogens’ adhesion, with inhibition rates of 42–70%, not unlike those obtained when used in combination with cell-free supernatant (48–63%). Among non-living BC17 derivatives, CFS-BC17 proved to be more active than HK-BC17 (inhibition rates of 34–48% and 29–31%, respectively), thus showing an opposite trend compared to what was observed with bifidobacteria.

On the other hand, the concomitant incubation of viable BC17 or viable+CFS-BC17 with enteropathogens significantly reduced the adhesion of *L. vaginalis* BC17 to Caco-2 cells (Figure 6). Indeed, when ETEC, *S. enterica*, and *Y. enterocolitica* were added to cells along with viable BC17, *L. vaginalis* BC17 adhesion decreased from 2.6 adherent BC17 per Caco-2 cell to 0.9, 1.8 and 1.4 adherent BC17 per Caco-2 cell, respectively. When viable BC17 was present in association with its supernatant (viable+CFS-BC17) and together with enteropathogens, the adhesion of *L. vaginalis* BC17 was found to be 1.3–2.3, corresponding to a reduction of 54–74% compared to the adhesion of viable+CFS-BC17 alone.

### 3.5. Anti-Inflammatory Properties of L. vaginalis BC17 and Its Derivatives

Two approaches were applied to investigate the anti-inflammatory properties of *L. vaginalis* BC17 fractions (viable BC17, CFS-BC17, HK-BC17, HK+CFS-BC17 and viable+CFS-BC17) on intestinal cells (Caco-2 and HT-29) and macrophages (RAW264.7).

#### 3.5.1. Protection of the Intestinal Cells from Inflammatory Stress

**Figure 8:**
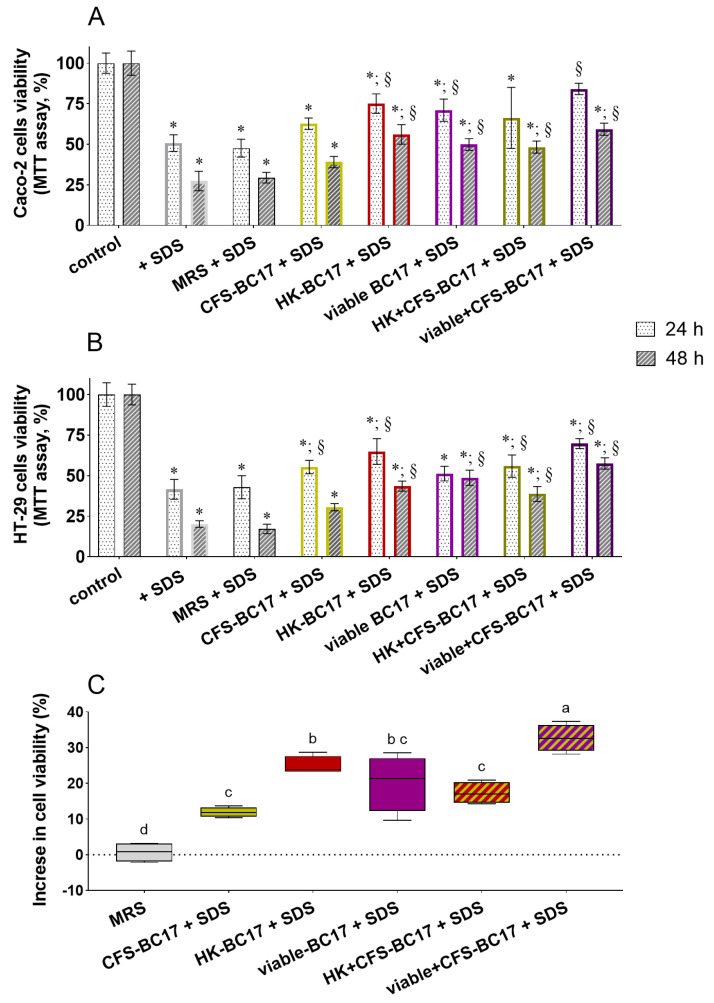
Protective effect of *L. vaginalis* BC17 and its derivatives on Caco-2 and HT-29 cells exposed to inflammatory stress. Residual cell viability after exposure to SDS 0.05% for 24 h and 48 h evaluated by MTT assay on (**A**) Caco-2 cells; (**B**) HT-29 cells. Results are expressed as percentages compared to the control (100%) (mean ± SD, *n* = 3) * *p* < 0.05. Significant differences calculated with respect to cells exposed to SDS 0.05% (+SDS) are also reported (§ *p* < 0.05). (**C**) Boxplot of the protective effects of *L. vaginalis* BC17 fractions, reported as the increase in cell viability (%) compared to +SDS (dotted line). Each box represents the interquartile range (25–75th percentile). Lines within the boxes indicate the median values of the samples. The extremes of the bars indicate the minimum and maximum values, respectively. Statistically significant differences between the samples are indicated by different letters (*p* < 0.05).

In order to evaluate the protective effect of *L. vaginalis* BC17 fractions, Caco-2 and HT-29 cells were exposed to 0.05% SDS, an irritant agent to induce an inflammatory state [35,40]. This reduced Caco-2 cell viability by 49% (24 h) and 73% (48 h) (Figure 8A), and HT-29 cell viability by 58% (24 h) and 80% (48 h) (Figure 8B), compared to cells not exposed to SDS (control). Overall, the concomitant treatment of Caco-2 and HT-29 cells with *L. vaginalis* BC17 fractions resulted in a significant reduction in the damage caused by SDS, although there were differences among *L. vaginalis* BC17 fractions. Specifically, the cell-free supernatant was the least protective, improving the viability of only HT-29 cells after 24 h of incubation (+14%, *p* < 0.05), while the presence of MRS did not exert any protective effect. *L. vaginalis* BC17 cells (HK-BC17 and viable-BC17), on the other hand, efficiently counteracted the loss of viability caused by SDS, as highlighted in the boxplot in Figure 8C. HK-BC17 significantly improved the viability of both Caco2 and HT-29 cells after 24 h (+23–24%) and 48 h of incubation (+23–29%). Viable BC17 was only slightly less effective than its heat-killed counterpart, significantly increasing Caco-2 cell viability by 20% (24 h) and 23% (48 h), and HT-29 cell viability by 28% after 48 h of incubation. The combination of HK-BC17 and CFS-BC17 resulted in intermediate activity between the two individual fractions, leading to a 16–21% increase in cell viability. Notably, the combination of viable BC17 and CFS-BC17 showed the best profile (Figure 8C, *p* < 0.05), as it was able to increase cell viability by 27–38%, effectively restoring Caco-2 cell viability after 24 h of incubation to the level of the control cells not exposed to SDS.

#### 3.5.2. Reduction of Nitric Oxide Production in Macrophages

**Figure 9:**
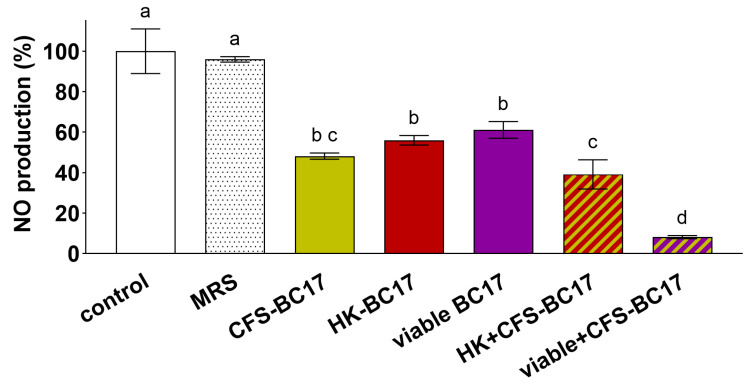
Inhibitory effect of *L. vaginalis* BC17 and its derivatives on NO production in LPS-induced macrophages. Results are presented as percentages relative to the control (untreated cells) (means ± SD, *n* = 3). Statistically significant differences between the samples are indicated by different letters (*p* < 0.05).

The immunomodulating activity was also investigated in terms of capacity to reduce a mediator of the inflammatory process, namely NO, in LPS-induced RAW264.7 macrophages [41,42]. The results are presented in Figure 9. Both viable BC17 and non-living derivatives (CFS-BC17 and HK-BC17) efficiently reduced NO production by 40% (viable BC17) and 52% (CFS-BC17) (*p* < 0.05), with no significant differences among these fractions. The combination of HK-BC17 and CFS-BC17 reduced NO production by 61%, making it more anti-inflammatory than HK-BC17 alone (44%). The simultaneous incubation with viable BC17 and its supernatant appeared to have an additive effect, reducing NO production by 92% and confirming the greater anti-inflammatory potential of viable+CFS-BC17 observed in the previous section.

### 3.6. Formulation of L. vaginalis BC17 in Fast-Disintegrating Tablets

The development of a suitable dosage form for the oral delivery of *L. vaginalis* BC17 was pursued with the aim of enhancing the stability and shelf life of the functional ingredients as well as providing a convenient mode of administration.

#### 3.6.1. Selection of Tablet Composition

The images reported in Table 1 show that the use of MX at 30% (*w*/*w*) alone allowed the formation of compact and uniform tablets; however, they were highly brittle and broke easily when handled. Conversely, using undiluted CFS-BC17 alone did not allow for the formation of properly shaped tablets: the freeze-dried product was sticky, forming a non-uniform thin matrix covering the internal surface of the blister pocket, and failed to remain within the mould. Nevertheless, this observation suggested that CFS-BC17 might act as a binder/plasticizer, improving the compactness and structural integrity of the fragile MX-based tablets. Blending different concentrations of MX with diluted or undiluted CFS-BC17 did not affect MX solubility in the supernatant but revealed that increasing MX concentration enhanced tablet compactness. However, despite the presence of CFS-BC17, tablets containing 10 and 20% MX were excessively friable and partially adhered to the blister pack cavity. Moreover, regardless of MX concentration, the use of undiluted CFS-BC17 improved surface uniformity, resulting in more compact tablets. Data in Table 1 demonstrate that tablets formulated with the combination of the two excipients exhibited similar diameters and thicknesses. Nevertheless, a slight but significant difference in diameter was observed between tablets containing 30% MX and those with 10% MX, both in the presence of diluted and undiluted CFS-BC17 (*p* < 0.05). As expected, the tablets’ weight increased gradually with higher MX concentrations and was statistically different (*p* < 0.05) between tablets prepared with diluted or undiluted CFS-BC17 at the same MX concentration. Based on their superior compactness, resistance to handling, and uniformity, the final formulations, comprising either viable (Tab viable+CFS-BC17) or heat-killed cells (Tab HK+CFS-BC17) were prepared using 30% (*w*/*w*) MX solubilized in undiluted CFS-BC17.

#### 3.6.2. Technological Characterization of Tablets

Table 2 summarizes the main features of the BC17-containing tablets, which did not significantly differ (*p* > 0.05) in terms of diameter, thickness, and weight from one another. Although the pH of CFS-BC17 was adjusted to 6.5 prior to formulation, the pH of the dispersed tablets was measured to establish whether MX or *L. vaginalis* BC17 cells influenced it. Both selected tablet types exhibited a pH close to 6.8, aligning with the physiological pH of the buccal cavity [43]. The content uniformity was checked on the final product by a turbidimetric approach, as a critical quality control assessment. The results indicated suitable uniformity across different batches, as demonstrated by the low standard deviation (Table 2). Furthermore, since Tab viable+CFS-BC17 and Tab HK+CFS-BC17 were prepared with the same cell concentration, the absorbance (ABS) values measured for the two formulations were not statistically different (*p* > 0.05). The ABS values obtained for Tab Viable+CFS-BC17 and Tab HK+CFS-BC17 did not differ, since heat-killing did not result in cell lysis and therefore, viable or inactivated cells produced comparable turbidities.

**Figure 10:**
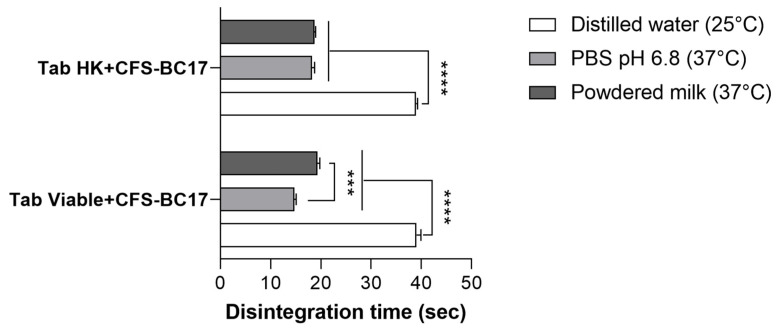
Disintegration time of the selected tablets in relevant media. Data are reported as mean ± SD, *n* = 3. ***p < 0.001; **** *p* < 0.0001.

To simulate various conditions, the disintegration of the tablets was evaluated in different media: water at room temperature, phosphate buffer with pH and temperature similar to human saliva, and warm powdered milk. Hence, fast-disintegrating tablets can be dispersed in a glass of water or directly placed on the tongue. Moreover, considering the pediatric application, probiotics and postbiotics can be administered via a baby bottle with milk. As shown in Figure 10, the selected tablets fully disintegrated within 39 s in water, and, notably, disintegration times in PBS and milk were significantly faster (*p* < 0.0001), occurring within 19 s or less, likely due to the temperature difference. Interestingly, for Tab viable+CFS-BC17, disintegration in PBS was significantly faster (*p* < 0.001) compared to powdered milk.

#### 3.6.3. Technological Stability of Tablets and Contamination Check

Physical parameters of both Tab viable+CFS-BC17 and Tab HK+CFS-BC17 were evaluated once a month (T1 = 30 days, T2 = 60 days, T3 = 90 days). Comparing size and weight measurements at T1, T2, and T3, the tablets appeared stable, and no significant differences were observed (*p* > 0.05) (Table 3).

As oral non-aqueous formulations, the developed fast-disintegrating tablets belong to non-sterile dosage forms. As a result, they should meet the acceptance criteria for aerobic bacteria (10^3^ CFU/g or CFU/mL), yeasts, and molds (10^2^ CFU/g or CFU/mL) total count, as well as the absence of specific microorganisms, particularly *Escherichia coli* [44]. Therefore, the selected tablets were also monitored for microbiological quality. Three different media were tested: NA and TSA plates to evaluate bacterial contamination, and SDA plates to evaluate fungal presence. At all considered time points, no microbial contamination was observed. These results indicate that any potential microbial presence was below the sensitivity limit of the technique (<10 CFU/mL) and, consequently, also met the acceptance criteria for oral formulations.

#### 3.6.4. Survival of *L. vaginalis* BC17

**Figure 11:**
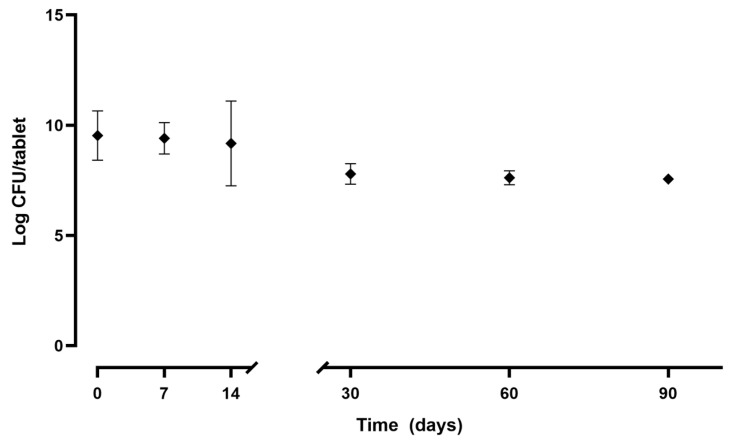
Viability of *L. vaginalis* BC17 cells formulated in Tab viable+CFS-BC17. Viability, expressed as LogCFU/tablet, was measured immediately after freeze-drying (0 days) and at different time points (0, 7, 14, 30, 60, 90 days) (mean ± SD, *n* = 2).

The viability of *L. vaginalis* BC17 in Tab viable+CFS-BC17 was monitored for up to 90 days, and results are shown in Figure 11. Firstly, the survival rate after freeze-drying was evaluated: up to 77% of *L. vaginalis* BC17 survived the technological process (3.8 × 10^9^ CFU/tablet at time 0 compared to 5.0 × 10^9^ CFU before freeze-drying).

Tablets were then stored at room temperature in a desiccator, and the stability in terms of viability was investigated. Comparing the number of viable cells per tablet in the first three time points (0, 7, 14 days), no remarkable variations were observed (*p* > 0.05). Between 14 and 30 days, a significant (*p* < 0.05) decrease in viability was noticed: viable cell counts dropped from 1.9 × 10^9^ CFU/tablet (at 14 days) to 6.3 × 10^7^ CFU/tablet (at 30 days). At the last two time points (60 and 90 days), *L. vaginalis* BC17 viability appeared stable, reaching a concentration of 3.7 × 10^7^ viable cells per tablet at the end of the stability study.

#### 3.6.5. Functional Characterization of Tablets and Stability

Tablets containing cell-free supernatant combined with heat-killed (Tab HK+CFS-BC17) or living cells (Tab viable+CFS-BC17) were tested to ensure that the formulations retained the functional properties of *L. vaginalis* BC17 and its derivatives, both immediately after preparation (T0) and after 3 months of storage (T3) (Figure 12A–D).

Importantly, no negative effects were observed on the viability of Caco-2 and HT-29 cells, investigated by the MTT assay and erythrosine B exclusion test, as extensively reported in Figure S1 of Supplementary Materials and summarized in Figure 12A. Conversely, Tab HK+CFS-BC17 and Tab viable+CFS-BC17 (T0) increased intestinal cell viability by up to 59% and 76%, respectively, aligning with the effects observed for non-formulated HK+CFS-BC17 (viability increase up to 50%) and viable+CFS-BC17 (viability increase up to 62%) (*p* < 0.05).

Additionally, tablets significantly enhanced the capacity of bifidobacteria strains (i.e., *B. bifidum* DSM 20082, *B. adolescentis* DSM 20086, *B. infantis* DSM 20090, *B. breve* DSM 20091, and *B. longum* DSM 20219) to adhere to Caco-2 cells (Figure 12B and Figure S2). Specifically, Tab viable+CFS-BC17 promoted *Bifidobacterium* spp. adhesion more efficiently (+58–183%, T0) than Tab HK+CFS-BC17 (+30–126%, T0), consistent with previous findings for non-formulated viable+CFS-BC17 (+56–174%) and HK+CFS-BC17 (+21–102%) (Figure 12B).

Simultaneously, formulations reduced the adhesion of enterotoxigenic *E. coli* to Caco-2 cells, from 4.0 adherent bacteria/cell (control) to 2.9 adherent bacteria/cell (Tab HK+CFS-BC17, T0) and 2.2 adherent bacteria/cell (Tab viable+CFS-BC17, T0) (Figure S2), thereby also retaining their anti-pathogenic functionality.

Regarding anti-inflammatory properties, protective effects on Caco-2 and HT-29 cells under SDS-induced stress were observed in the presence of Tab HK+CFS-BC17 and Tab viable+CFS-BC17 (Figure 12C), as they increased cell viability by 26–30% (T0) and 23–56% (T0), respectively. In particular, Tab viable+CFS-BC17 was slightly more protective than Tab HK+CFS-BC17 since it completely restored Caco-2 cell viability after 24 h and 48 h of SDS exposure (Figure S3).

Tab viable+CFS-BC17 also showed excellent anti-inflammatory properties in an LPS-induced macrophages model. Indeed, it reduced NO production by 93% (T0), compared to a 63% reduction observed with Tab HK+CFS-BC17, in line with the effects of non-formulated *L. vaginalis* BC17 fractions (Figure 12D). Importantly, all these properties were confirmed when tablets were tested 3 months after production (T3), with no significant loss of functionality compared to T0.

## 4. Discussion

Several lines of evidence support the use of probiotics, prebiotics, and synbiotics to shape the gut microbiota in C-section delivered, preterm and formula-fed newborns, to restore beneficial microflora dominated by bifidobacteria. A close interconnection has been observed between the mother’s vaginal microbiota and the development of the infant’s gut microbiota and immune system maturation [8,11]. The physiological relevance of this interaction/contamination between ecosystems supports and justifies the choice of a vaginal *Lactobacillus* strain for its implications and applications in gut health, particularly in pediatrics.

*L. vaginalis* BC17 is a vaginally isolated strain which exhibits pronounced bifidogenic activity, demonstrated for both cell culture supernatant and heat-killed cells, paving the feasibility of using non-living derivatives as biotherapeutics [16,17]. In the present study, the health-promoting activities of *L. vaginalis* BC17 and its derivatives have been further explored. Despite the potential benefits of probiotics and postbiotics, safety remains a paramount aspect to be considered when proposing a novel strain for human consumption [45]. In this regard, the harmful presence of antibiotic resistance and other virulence factors [46] has been previously excluded by genome analysis of *L. vaginalis* BC17 [17].

In vitro and in vivo tests have excluded genotoxicity for several lactobacilli species most employed as probiotics, including *Lacticaseibacillus paracasei* [47], *Lacticaseibacillus rhamnosus* [48], *Lactiplantibacillus plantarum* [49], and *Limosilactobacillus fermentum* [50]. To date, no information is available regarding the vaginal species *L. vaginalis*. Considering that toxicity is primarily due to the production of metabolites and metabolism byproducts [51], we evaluated the genotoxicity of two derivatives of *L. vaginalis* BC17: the cell-free supernatant, which contains the pool of substances produced during fermentation, and heat-killed cells, which are non-viable cells comprising structural components and intracellular metabolites. Importantly, in vitro mammalian cell micronuclei test, revealed that *L. vaginalis* BC17 derivatives were not mutagenic, as they did not induce chromosomal aberrations.

In the view of oral intake, the safety of *L. vaginalis* BC17 and its derivatives was deeper investigated on cell lines of intestinal origin with distinct phenotypes (i.e., Caco-2 and HT-29 cells) [37]. Neither living *L. vaginalis* BC17 nor its derivatives (CFS-BC17 and HK-BC17) compromised the viability and metabolic activity of intestinal cells. These results are in line with previous studies that demonstrated the absence of cytotoxicity of other lactobacilli species, i.e., *L. rhamnosus, L. plantarum* and *L. fermentum*, toward Caco-2 [52,53] and HT-29 cells [54].

By contrast, our results showed that *L. vaginalis* BC17 and its derivatives enhanced the viability of intestinal cells. In this regard, many probiotics have been reported to stimulate the proliferation of intestinal cells, thereby contributing to the maintenance and turnover of the intestinal mucosa barrier [55]. For instance, certain strains of *Limosilactobacillus reuteri*, when co-incubated with intestinal cells, have been found to enhance enterocyte proliferation by inducing the STAT3 and the Wnt/β-catenin pathway [56]. Proliferative and maturation outcomes on intestinal cells were also observed following treatment with a postbiotic of *L. reuteri* DS0384, specifically cell-free supernatant, and the effects seem to be ascribed to metabolites involved in glutamine metabolism and the urea cycle [57]. Additionally, lactobacilli produce lactate and short chain fatty acids (SCFAs), particularly butyrate, which serve as energy sources for colonocytes, thus improving epithelial barrier and promoting intestinal cells proliferation [56,58]. Other components of lactobacilli cell wall, such as exopolysaccharides (EPS) and peptidoglycan, may also favour the intestinal cell viability [59], as supported by the observation that heat-killed *L. vaginalis* BC17 exerted a stimulating activity only slightly lower than that of living cells. Bioactive peptides secreted into the *milieu* or present in CFS-BC17 may also support intestinal cell viability. In this regard, metabolomic analysis of CFS-BC17 [16] revealed a high abundance of several amino acids, including alanine, aspartate, glutamate, proline, leucine, isoleucine, and valine, which are known to contribute to epithelial cell function and proliferation [60,61]. Aromatic amino acids such as tryptophan and tyrosine were also abundant and can be converted into bioactive metabolites with immunomodulatory and mitogenic properties [62]. Moreover, dietary amino acids such as methionine and cysteine have been shown to enhance gut barrier integrity, support antioxidant defenses, and promote epithelial cell proliferation, supporting the potential role of sulfur-containing amino acids detected in CFS-BC17 in sustaining epithelial viability [63,64].

The capacity of a probiotic strain to temporarily colonize the colon and survive in the gut microenvironment is desirable to ensure a prolonged health-promoting effect on the host. *L. vaginalis* BC17 showed good resistance to gastric acidity and moderate ability to adhere to the Caco-2 cells monolayer, as already reported by D’Alessandro et al. [27], and low adhesion to HT-29 cells that, notably, increased at prolonged incubation times.

Despite the high complexity of the intestinal environment and the inherent limitations of a cell model, these results, along with the high viability rate of *L. vaginalis* BC17 in co-incubation with Caco-2/HT-29 cells, are encouraging and they support the use of this strain as a probiotic.

The consumption of probiotics and postbiotics can modulate gut microbiota composition and help mitigate gut dysbiosis [65]. In previous works [16,17] we found that *L. vaginalis* BC17 derivatives reduced enteropathogens’ planktonic growth and their biofilms. The anti-pathogenic potential of *L. vaginalis* BC17 viable cells and derivatives was further demonstrated in the present manuscript by the evidence that they significantly reduced the adhesion of enteropathogens’ adhesion to Caco-2 cells. This is in line with other studies reporting anti-adhesive properties of viable lactobacilli and heat-killed cells against a plethora of relevant intestinal pathogens [66,67,68,69,70]. Lactobacilli can compete with pathogens for adhesion sites, as they possess surface adhesins similar to those of bacterial pathogens [66]. Considering the ability of *L. vaginalis* BC17 to adhere to Caco-2 cells, it is likely that competitive exclusion with pathogens for adhesion sites is at least partially responsible for the anti-adhesive effect. However, in the presence of enteropathogens, the adhesion of *L. vaginalis* BC17 turned out to be slightly reduced, suggesting that it could co-aggregate with the pathogens in the culture medium, resulting in a decreased adhesion of both. It has also been reported that molecules on the surface of probiotic cells (such as peptidoglycan, lipoteichoic acid, cell wall polysaccharide, and S-layer proteins) can bind to pathogens and consequently reduce their adhesion capacity to intestinal cells [71,72]. This is consistent with the observation that also heat-killed BC17 impaired the adhesion of enteropathogens to Caco-2 cells, albeit less efficiently than viable-BC17. Additionally, the cell-free supernatant of *L. vaginalis* BC17 antagonized pathogens’ adhesion, likely due to the action of secreted biomolecules with anti-adhesive properties (e.g., biosurfactants and EPS).

Physiologically, another way in which probiotics can strengthen the intestinal barrier and indirectly prevent pathogen invasion, is through the stimulation of beneficial microflora, mainly represented by bifidobacteria. It’s noteworthy that the adhesion of *L. vaginalis* BC17 to Caco-2 cells increased when co-incubated with *Bifidobacterium* spp., suggesting that *L. vaginalis* BC17 and bifidobacteria could cooperate and interact with each other, promoting the adhesion of both. It is known from clinical trials that a combination of *L. acidophilus*, *L. rhamnosus*, *L. plantarum*, and *B. bifidum* can modulate gut microbiota by boosting the enrichment of *Bifidobacterium* and *Lactobacillus* [73]. In the present paper, we reported for the first time that a vaginal *Lactobacillus* strain and its derivatives were able to enhance the adhesion of *Bifidobacterium* spp. to intestinal cells in an in vitro model, thereby confirming the bifidogenic properties previously observed in simple planktonic and biofilm experimental setups [16,17]. The mechanisms responsible for this cooperation are still unknown and will require further efforts to be elucidated. However, metabolites such as EPS may facilitate the adhesion of both bifidobacteria and the producing strain itself to intestinal cells, acting as prebiotics [74,75]. In this regard, genes related to the EPS synthesis pathway (e.g., sugar transferase cluster) have been found in the *L. vaginalis* BC17 genome [17], and it can be speculated that EPS molecules contribute to the observed bifidogenic behaviour. In another work, we demonstrated that EPS secreted by vaginal *L. crispatus* and *L. gasseri* promote the biofilm formation of several lactobacilli, including the producing strain [76]. This aspect could open new research perspectives, further supported by the observation that BC17 supernatant, in addition to stimulating bifidobacteria adhesion, also promoted the adhesion of *L. vaginalis* BC17 itself. Along with EPS, other cell wall components (such as peptidoglycan, lipoteichoic acids, and S-layer proteins) can influence the expression of adhesion molecules on intestinal epithelial cells, such as mucins and glycoproteins, which help protect against harmful bacteria while simultaneously making the cells more receptive to beneficial bacteria [77]. In fact, even heat-killed BC17 effectively stimulated the adhesion of bifidobacteria to Caco-2 cells, highlighting that the presence of living probiotics is not strictly necessary for a beneficial outcome.

This holds true also when considering the anti-inflammatory activity of both *L. vaginalis* BC17 and its derivatives. In fact, they protected intestinal cells (Caco-2 and HT-29) from damage caused by oxidative stress induced by SDS, suggesting a potential in preventing exacerbated inflammatory responses that, in turn, could contribute to the chronicization of pathologic states.

To date, many *Lactobacillus* species and postbiotics have been shown to alleviate gut inflammation and oxidative stress both in vitro and in rodent models of DSS-induced colitis, but no information is yet available for *L. vaginalis* species. In this study, we utilized two cell lines with distinct characteristics: Caco-2 cells are known to form tight junctions (TJs), whereas HT-29 cells fail to generate stable TJs but are mucus-producing cells [78]. Considering that *L. vaginalis* BC17 provided protection for both cell lines, we can infer that this strain and its derivatives may act through different mechanisms to restore barrier integrity, such as enhancing the TEER (transepithelial electrical resistance) levels and modulating the TJ proteins. Another possible mechanism is the promotion of mucus secretion, particularly through the upregulation of *MUC2* gene expression in goblet cells. Similar activities have been reported for the multi-strain probiotic VSL#3 [79,80] and *L. rhamnosus* GG [81]. It is also likely that the ability of *L. vaginalis* BC17 to promote the turnover of Caco-2 and HT-29 cells, as discussed above, contributes to its protective effect. These results, along with the capacity to reduce the production of an inflammatory mediator (i.e., nitric oxide) in RAW264.7 cells, suggest that *L. vaginalis* BC17 and its derivatives may also be useful in alleviating inflammatory states. It should be highlighted that the anti-inflammatory activity of viable and heat-killed BC17 is comparable, supporting the use of postbiotics as a valid alternative to the live strain. Viable cells exhibited slightly greater activity than heat-killed cells in terms of competition with pathogens and stimulation of bifidobacteria, likely because these activities are linked to cell wall components that may be susceptible to heat treatment [66,71]. With the aim of further investigating the interaction of *L. vaginalis* BC17 with the gut-associated immune system, we plan to carry out targeted studies in the future to evaluate the modulation by this strain of the expression of key cytokines involved in the cross-talk between the microbiota and the host, such as IL-10, TNF-α, IL-6, and others.

To maximize the beneficial effects of viable or heat-killed cells and supernatant of *L. vaginalis* BC17, we developed oral formulations that combine both components. Since the use of living cells still poses safety concerns for vulnerable people (e.g., immunocompromised individuals, preterm infants) [82], the alternative formulation, containing the heat-killed cells, appears extremely safe while maintaining its functionality. Moreover, postbiotic preparations also offer some pharmaceutical advantages over their living counterparts, since they are easier to standardize, transport, and store. In this study we proposed an oral solid formulation in the form of freeze-dried tablets for the delivery of *L. vaginalis* BC17 living cells and/or its postbiotics. Even though freeze-drying is an easily scalable and convenient technological approach to obtain a solid matrix, it exposes probiotics to significant stresses, such as cold stress, crystallization, osmotic pressure, and desiccation, which can severely impact cell survival by compromising membrane integrity, disrupting metabolism, and impairing enzymatic activity [83,84]. To minimize structural damage to bacteria and preserve probiotic and postbiotic properties during freeze-drying, sugar-based cryoprotectants like maltodextrin and sucrose are commonly used [85]. Particularly, maltodextrins act as crystallization inhibitors, stabilizers, carriers, bulking agents and exhibit anti-collapse properties, preventing structural shrinkage during freeze-drying [86]. Instead of including an additional excipient in the formulation as binder/plasticizer, it turned out that the BC17 supernatant can serve this function, likely due to some byproducts of the microbial metabolism, namely amino acids, organic acids, monosaccharides, ketones, and alcohols [26]. Therefore, the produced tablets stand out for the dual role of BC17 supernatant, serving as both bioactive substance and excipient, and also ensuring cost-effective and scalable production through a straightforward development process that minimizes waste. The employed formulation approach sustained the viability of the formulated probiotic as well as its biological activity and that of the postbiotics; moreover, it allowed the development of tablets whose technological features were consistent among different batches, ensuring high reproducibility. Besides, what makes these tablets particularly advantageous is their versatility, in terms of appropriateness for patients of all ages. In fact, freeze-drying of maltodextrin solutions produces an amorphous porous network which rapidly disperses in water, thus resulting in fast-disintegrating tablets [86]. These can be assumed as orodispersible tablets by adults and patients having difficulties with swallowing tablets or hard gelatin capsules [87], but they are also suitable for pediatric applications, since tablets freely disintegrated in less than 1 min in different media, such as water and milk. Specifically, as regards pediatric administration of oral dosage forms, the guidelines released in 2011 by the European Medicines Agency, recommend liquid dosage forms for patients below the age of 2, while for patients over 2 years old, orally disintegrating tablets can also be administered [88]. As a result, the former are supposed to receive the prebiotic/postbiotic after tablet disintegration in powdered milk, and the latter can either solubilize the tablet in a liquid or take the FDT directly. Additionally, the developed FDT were thin and within the size range of the 15 most prescribed oral solid dosage forms in the pediatric population [88]. Moreover, considering the size and the low weight (0.18 g) of our tablets, the volume that they cover in the oral cavity could be extremely restricted. Finally, they dissolve in a liquid form in 19 s in the mouth, limiting the potential choking risk. Tablets also featured a noteworthy shelf life, with their physical properties and health-promoting effects being preserved after three months of storage at room temperature. However, a decrease in *L. vaginalis* BC17 viability was observed from 2 months onward, in agreement with previously reported findings related to probiotics formulations stored at room temperature [89,90,91]. Nevertheless, none of the health-promoting activities exerted by living *L. vaginalis* BC17 were compromised after three months of storage, meaning that a reduction in the number of viable cells did not result in a loss of functionality.

Future studies aim to investigate the adaptation of *L. vaginalis* BC17 within the intestinal ecosystem, as well as the modulation exerted by the strain and its formulations on the composition and metabolism of gut microbiota, using in vitro and in vivo gut models representative of both pediatric and adult populations.

## 5. Conclusions

Our findings offer a promising solution to the issue of insufficient bifidogenic stimuli in infants born via caesarean section and formula-fed, suggesting the use of *L. vaginalis* BC17 and its derivatives as biotherapeutics to promote bifidobacterial colonization in early childhood. Keeping in mind that adults suffering from dysbiosis and inflammatory conditions can also benefit from interventions based on probiotics (live beneficial microorganisms) or postbiotics (non-viable derivatives of beneficial microorganisms), versatile formulations have been successfully developed for both infant and adult supplementation. The perspective is to improve intestinal and immune health and overall well-being, with significant social implications, particularly in pediatric healthcare. To validate the clinical relevance of our biotherapeutic solution, further investigations will be necessary, including in vivo studies.

## Figures and Tables

**Table 1:** Tablets’ characteristics as a function of their composition. Tablets were obtained using either maltodextrin (MX) or diluted (dil 1:2)/undiluted (ud) cell-free supernatant from BC17 (CFS-BC17) or the combination of the two. When the excipients used did not allow for the formation of a tablet, size and weight could not be measured, thus NM (not measured) is indicated in the table. Data are reported as mean value ± SD, *n* = 6.

Tablet’s Composition	Diameter (mm)	Thickness (mm)	Weight (g)	Image
MX 30%	12.97 ± 0.14	3.72 ± 0.10	0.17 ± 0.01	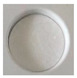
MX 10% + CFS-BC17 dil 1:2	12.93 ± 0.19	3.85 ± 0.24	0.08 ± 0.00	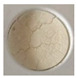
MX 10% + CFS-BC17 ud	12.89 ± 0.17	3.87 ± 0.29	0.09 ± 0.00	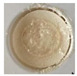
MX 20% + CFS-BC17 dil 1:2	13.23 ± 0.34	3.93 ± 0.29	0.14 ± 0.00	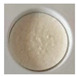
MX 20% + CFS-BC17 ud	13.06 ± 0.16	3.95 ± 0.29	0.15 ± 0.01	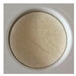
MX 30% + CFS-BC17 dil 1:2	13.29 ± 0.27	4.05 ± 0.16	0.18 ± 0.01	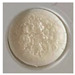
MX 30% + CFS-BC17 ud	13.28 ± 0.30	4.02 ± 0.14	0.18 ± 0.00	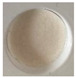

**Table 2:** Characteristics of newly prepared tablets (T0) as a function of their composition. Tablets containing viable or heat-killed (HK) cells were obtained using 30% maltodextrin and undiluted cell-free supernatant from BC17 (CFS-BC17). Diameter, thickness, weight, pH, and content uniformity are reported for each tablet. Data are reported as mean value ± SD, *n* = 3.

Tablet	Diameter (mm)	Thickness (mm)	Weight (g)	pH	Content Uniformity (ABS)
Viable+CFS-BC17	13.35 ± 0.22	4.33 ± 0.25	0.18 ± 0.00	6.89 ± 0.17	0.24 ± 0.00
HK+CFS-BC17	13.42 ± 0.18	4.30 ± 0.27	0.18 ± 0.01	6.77 ± 0.03	0.24 ± 0.01

**Table 3:** Technological features of tablets over time. Diameter, thickness, and weight were measured at T1 (30 days), T2 (60 days), and T3 (90 days). Data are reported as their mean value ± SD, *n* ≥ 5.

Tablet	T1			T2			T3		
	Diameter (mm)	Thickness (mm)	Weight (g)	Diameter (mm)	Thickness (mm)	Weight (g)	Diameter (mm)	Thickness (mm)	Weight (g)
Viable+CFS-BC17	13.47 ± 0.44	4.27 ± 0.20	0.18 ± 0.01	13.54 ± 0.43	4.21 ± 0.23	0.18 ± 0.01	13.85 ± 0.22	4.21 ± 0.23	0.18 ± 0.01
HK+CFS-BC17	13.46 ± 0.37	4.27 ± 0.29	0.18 ± 0.01	13.62 ± 0.41	4.21 ± 0.26	0.18 ± 0.01	13.94 ± 0.13	4.15 ± 0.23	0.18 ± 0.17

## Data Availability

All data are published in the AMS acta repository (https://doi.org/10.6092/unibo/amsacta/8051).

## Supplementary Materials

The following supporting information can be downloaded at: https://www.mdpi.com/article/10.3390/pharmaceutics17081011/s1. Figure S1: Viability of intestinal cells treated with tablets (Tab HK+CFS-BC17 and Tab viable+CFS-BC17) for 24 h and 48 h; Figure S2: Effects of tablets (Tab HK+CFS-BC17 and Tab viable+CFS-BC17) on the adhesion of *Bifidobacterium* spp. and ETEC to Caco-2 cells after 3 h of co-incubation; Figure S3: Protective effect of tablets (Tab HK+CFS-BC17 and Tab viable+CFS-BC17) on Caco-2 and HT-29 cells exposed to inflammatory stress.

## Abbreviations

The following abbreviations are used in this manuscript:

BHI	Brain Heart Infusion
CFS	Cell-Free Supernatant
CFU	Colony-Forming Unit
CP	CycloPhosphamide
EPS	ExoPolySaccharides
FBS	Fetal Bovine Serum
HK	Heat-Killed
LPS	LipoPolySaccharide
MMC	Mutagens Mitomycin C
MNi	MicroNuclei
MRS	de Man, Rogosa, and Sharpe broth
MTT	3-(45-diMethylThiazol-2-yl)-2,5-diphenylTetrazol
MX	MaltodeXtrin
NO	Nitric Oxide
NA	Nutrient Agar
OECD	Organisation for Economic Co-operation and Development
PBS	Phosphate Buffer Solution
PD	Population Doubling
RPD	Relative Population Doubling
SCFAs	Short-Chain Fatty Acids
SD	Standard Deviation
SDS	Sodium Dodecyl Sulfate
SEM	Standard Error of the Mean
TEER	TransEpithelial Electrical Resistance
TSA	Tripticase Soy Agar
VINB	VINBlastine
