# Supplementary material for: Functional and Safety Profile of Limosilactobacillus vaginalis and Development of Oral Fast-Disintegrating Tablets for Gut Microbiota Modulation
Source: Pharmaceutics. 2025 Aug 1;17(8):1011. doi: 10.3390/pharmaceutics17081011 (PMC12389607; doi:10.3390/pharmaceutics17081011)

Functional and Safety Profile of *Limosilactobacillus vaginalis* and Development of Oral Fast-Disintegrating Tablets for Gut Microbiota Modulation

Barbara Giordani [1], Federica Monti [1], Elisa Corazza [1], Sofia Gasperini [1], Carola Parolin [1], Angela Abruzzo [1], Claudio Foschi [2,3], Antonella Marangoni [2], Monia Lenzi [1], Barbara Luppi [1,*] and Beatrice Vitali [1,*]

Figure S1. Viability of intestinal cells treated with tablets (Tab HK+CFS-BC17 and Tab viable+CFS-BC17) for 24 h and 48 h. Tablets were tested immediately after the preparation (T0) and after 3 months of storage at room temperature (T3) on: (**A**) Caco-2 by means of MTT assay; (**B**) HT-29 by means of MTT assay; (**C**) Caco-2 by means of erythrosine B (dye) exclusion assay; (**D**) HT-29 by means of dye exclusion assay. Results are reported in percentages compared to the control (100%) (mean ± SD, n = 3), * $p < 0.05$. Significance between T0 and T3 of each formulation was also calculated, ns: not significant.

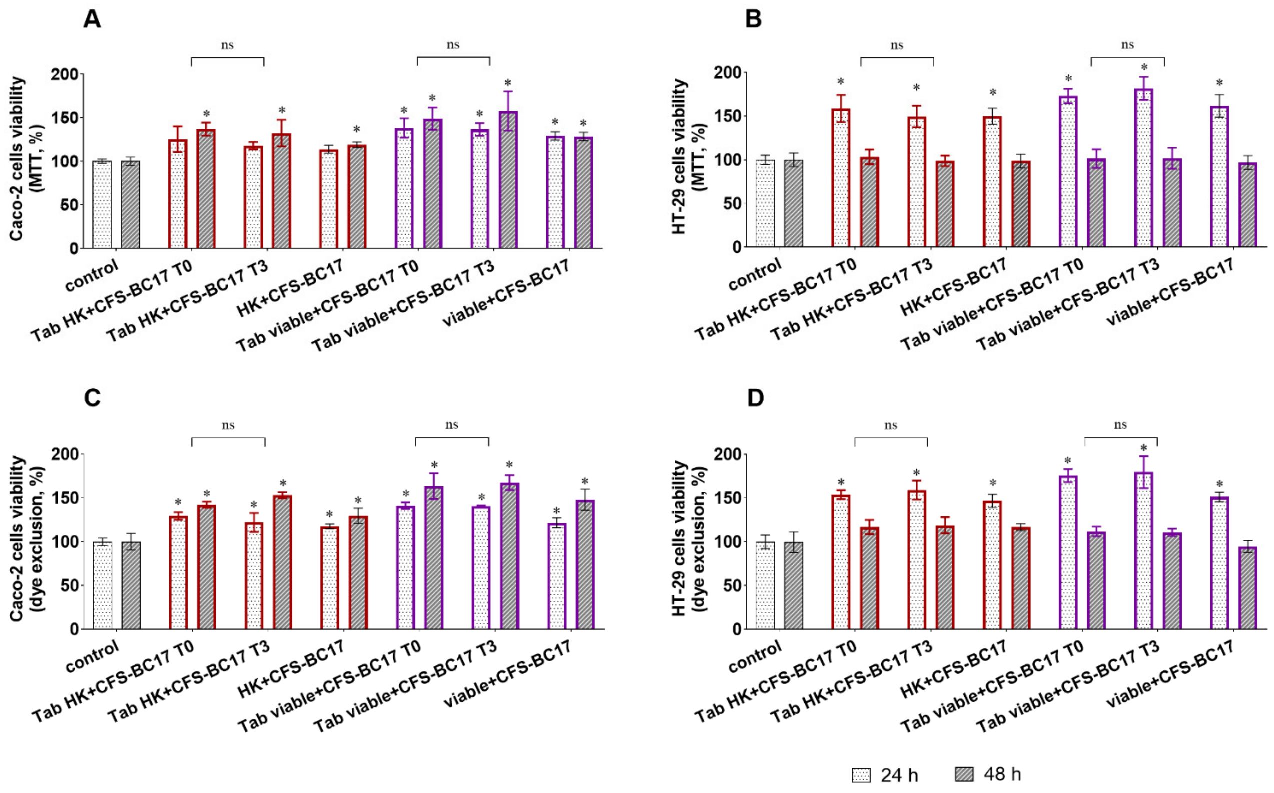

Figure S2. Effects of tablets (Tab HK+CFS-BC17 and Tab viable+CFS-BC17) on the adhesion of *Bifidobacterium* spp. and ETEC to Caco-2 cells after 3 h of co-incubation. Tablets were tested immediately after the preparation (T0) and after 3 months of storage at room temperature (T3) Results are reported as adherent bacteria per Caco-2 cell (mean ± SD, *n* = 3), * $p < 0.05$ (vs control). Significance between T0 and T3 of each formulation was also calculated, # $p < 0.05$, ns: not significant.

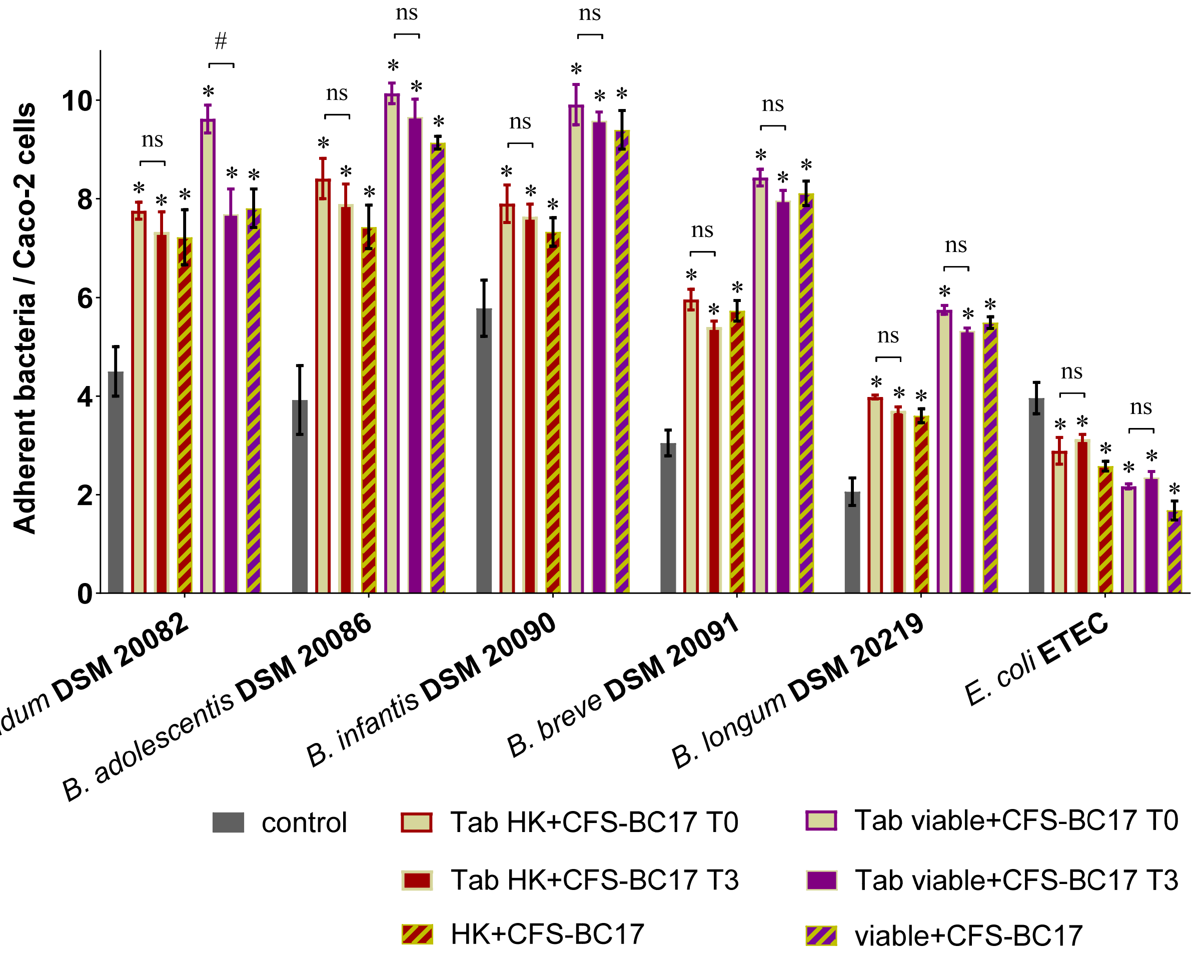

Figure S3. Protective effect of tablets (Tab HK+CFS-BC17 and Tab viable+CFS-BC17) on Caco-2 and HT-29 cells exposed to inflammatory stress. The residual cell viability after the exposure of SDS 0.05% for 24 h and 48 h was evaluated by MTT assay immediately after the preparation (T0) and after 3 months of storage at room temperature on: (**A**) Caco-2 cells; (**B**) HT-29 cells. Results are expressed as percentages compared to the control (100%) (mean ± SD, $n = 3$) * $p < 0.05$. Significant differences calculated with respect to cells exposed to SDS 0.05% (+ SDS) were reported as: § $p < 0.05$). Significance between T0 and T3 of each formulation was also calculated, ns: not significant.

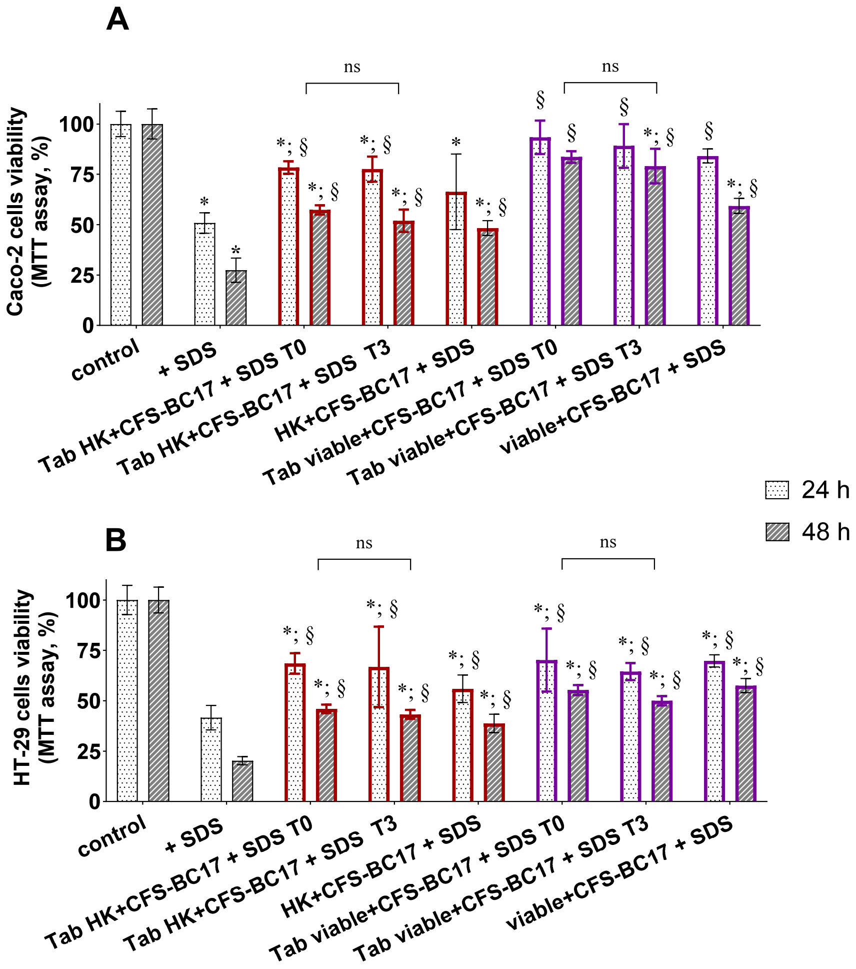

Supplement: Supplementary file 1 [file pharmaceutics-17-01011-s001.zip › pharmaceutics-3728508-supplementary.pdf]
